# Celebrating 50 years of fluorescence correlation spectroscopy (FCS): Advancing live-cell massively parallel FCS studies with photostable GFPs, mStayGold and StayGold/E138D^☆^

**DOI:** 10.1016/j.bbagen.2025.130809

**Published:** 2025-04-17

**Authors:** Sho Oasa, Borislav Stoyanov, Yuta Hamada, Stanko N. Nikolić, Aleksandar J. Krmpot, Akira Kitamura, Vladana Vukojević

**Affiliations:** a Department of Clinical Neuroscience, Center for Molecular Medicine, Karolinska Institutet, 17176 Stockholm, Sweden; b School of Science, Constructor University, 28759 Bremen, Germany; c Laboratory of Cellular and Molecular Sciences, Graduate School of Life Science, Hokkaido University, Sapporo 001-0021, Japan; d Institute of Physics Belgrade, University of Belgrade, 11080 Belgrade, Serbia; e Laboratory of Cellular and Molecular Sciences, Faculty of Advanced Life Science, Hokkaido University, Sapporo 001-0021, Japan

**Keywords:** Fluorescence correlation spectroscopy (FCS), and massively parallel FCS, mStayGold, Apparent brightness analysis, Glucocorticoid receptor, Homodimerization, Nucleocytoplasmic transport

## Abstract

More than 50 years after its inception, fluorescence correlation spectroscopy (FCS) remains a cornerstone technique for quantitative characterization of the cellular dynamics of molecules and their concentration and interactions in live cells. The enhanced green fluorescent protein (eGFP) has long been a preferred tag in live-cell FCS, valued for its brightness, photostability and lack of posttranslational modifications. However, low eGFP photostability limits measurement durations, posing challenges for studying dynamic cellular processes necessitating longer measurement time. Recent advancements in fluorescent protein engineering have yielded mStayGold and StayGold/E138D, two highly photostable monomeric GFP variants. In this study, we evaluate their performance in live cells and utility for FCS by quantifying glucocorticoid receptor (GR) homodimerization and nuclear import/export dynamics in live cells. Our study shows that both mStayGold and StayGold/E138D exhibit twice the brightness of eGFP, significantly enhancing the signal-to-noise ratio (SNR). Using massively parallel FCS (mpFCS) and two-foci cross-correlation to characterize the direction of GR nucleocytoplasmic transport along the nuclear envelope, we also confirm that these proteins show significantly improved photostability over eGFP.

## 1. Introduction

Fluorescence correlation spectroscopy (FCS), first proposed in 1972 as a method to study translational motion of molecules and measure chemical rate constants in a chemically reactive system at equilibrium through fluorescence intensity fluctuations of the fluorescent reaction product diffusing in and out of the focused laser beam [1], was formalized in 1974 in a series of seminal papers that laid out its theoretical and experimental foundation [2–4]. In 1993, integration of FCS with confocal microscopy revolutionized its potential for applications in biomedical research by enabling precise measurements in femtoliter volumes (in conventional confocal laser scanning microscopes the observation volume is (0.2–1) × 10^−15^ l) vastly improving the signal-to-noise ratio (SNR) [5] and enabling single-molecule sensitivity [6,7], which is of utmost relevance for measurements in live cells [8]. Over the following decades, innovations such as dual-colour fluorescence cross-correlation spectroscopy (FCCS) [9,10], two-photon FCS [11], scanning FCS [12], Förster resonance energy transfer FCS (FRET-FCS) [13], stimulated emission depletion FCS in sub-diffraction focal volumes (STED-FCS) [14], pin-hole array correlation imaging (PACI) [15], multipoint holographic FCS (MP-hFCS) [16], total-internal reflection FCS (TIR-FCS) [17], single-plane illumination FCS (SPIM-FCS) [18], inverse FCS (iFCS) [19], massively parallel FCS (mpFCS) [20–22], to name but a few, have been developed. These developments extended the FCS-toolbox and its applications for quantitative characterization of important determinants of the kinetics of biochemical reactions: the concentration of biomolecules and their fast intracellular dynamics in complex biological systems, *i.e.*, in live *ex vivo* organs [23], small organisms [24] and biological fluids for prospective disease diagnosis [25]. These endeavors build on the solid foundation laid by Prof. Rudolf Rigler, whose contribution has been indispensable for growing the FCS field, thereby advancing our understanding of molecular mechanisms underlying basic biological processes, and detailed mechanisms of action of approved [26] and prospective pharmacotherapeutics [27–29].

The application of FCS in biomedical studies is inseparable from the development of fluorescent proteins (FPs), imperative tools for live-cell fluorescence microscopy imaging and FCS. Their relevance is such that in 2008, the Nobel Prize in Chemistry was awarded to three laureates for their seminal work on discovering and developing the green fluorescent protein (GFP) [30], on which the brighter and the more photostable enhanced GFP (eGFP) is based.

FCS applications in biomedical research commonly rely on the use of eGFP as a genetically fused fluorescence tag for proteins of interest in live cells [31–35]. But FCS has also been used to characterize photophysical properties of fluorescent proteins [36–38], revealing their proneness to photobleaching. To account for this shortcoming, mathematical tools were developed to separate the fluorescence intensity fluctuations arising due to translational molecular motion through the observation volume element (OVE) from the steady decay in average fluorescence intensity due to photobleaching, known as the photobleaching correction [39–41]. While this correction method works well, it relies on the use of so-called raw FCS data, *i.e.*, photon counts, which necessitates working with large file sizes and is time consuming. To avoid difficulties arising due to fluorescent proteins’ photobleaching, alternative labeling strategies using engineered proteins such as the Halo and SNAP tags, are utilized to allow the use of photostable organic dyes. However, this approach is experimentally more cumbersome as it is necessary to establish the efficiency of organic dye labeling using the Halo/SNAP tags. Otherwise, protein concentration and the extent of protein-protein interactions (*e.g.*, under homo- or heterodimerization) will be underestimated.

Recently, Hirano et al. developed the photostable GFP dimer StayGold from *Cytaeis uchidae* [42]. StayGold is characterized by high photostability ($t_{1/2}^{\mathrm{StayGold}}=12,421\;s$: the time to reduce emission rate to half in live cells under wide-field illumination is about twenty-five (25) times longer than that of eGFP ($t_{1/2}^{\mathrm{eGFP}}=481\;s$), and of about 4-times higher brightness compared to that of eGFP under same conditions [42]. Ando et al. continued to construct the monomeric variant of StayGold, so called mStayGold [43], which retains the high photostability and high brightness of StayGold but is monomeric (molecular weight = 25.6 kDa). With the same aim, Ivorra-Molla et al. have developed the monomeric form of StayGold with a single E138D point mutation (StayGold/E138D) [44], and Zhang et al. developed the monomeric form of StayGold called mBaojin [45]. These bright and photostable GFPs allow for longer signal acquisition during FCS measurements, improving the signal-to-noise-ratio (SNR) and making complicated FCS data analysis redundant.

In this work, we have characterized two monomeric forms of StayGold, mStayGold and StayGold/E138D in live cells and assessed their photostability and brightness, as indicators of suitability for FCS measurements in conventional single-point FCS (spFCS) and massively parallel FCS (mpFCS). We have further quantitatively characterized ligand-stimulated homodimerization and nuclear import/export of the glucocorticoid receptor (GR) genetically fused with mStayGold. GR is a hormone-dependent transcription factor and a target for steroid pharmacotherapeutics [32]. In unstimulated cells, the GR is localized in the cytoplasm, bound into a large complex with other molecules. Upon ligand binding, this complex dissociates and GRs translocate to the cell nucleus [22], where they bind to the glucocorticoid response elements (GREs) in the genomic DNA and exert gene expression regulation function [46–49]. In parallel, ligand binding induces GR homodimerization and heterodimerization with other transcription factors for rewiring gene expression [50].

Our data clearly shows that increased brightnesses of mStayGold and StayGold/E138D allow us to reduce the excitation intensity, achieving the same SNR as when using eGFP, allowing at the same time longer time series data acquisition. This, in turn, improves the precision of autocorrelation curves’ fitting, as autocorrelation curves with less noise are obtained.

## 2. Materials and methods

### 2.1. Chemical reagents

The GR-specific synthetic ligand dexamethasone (Dex) was purchased from Sigma-Aldrich and used without any further purification. 2 mM stock solution of Dex was prepared by dissolving it in dimethyl sulfoxide (DMSO). The stock solution was dispensed, and aliquots were stored at −20 °C. For cell treatment, the stock solution was warmed to room temperature and diluted to 500 nM using phenol red-free FluoroBrite DMEM (Gibco) cell culture medium.

### 2.2. Plasmid DNAs

Plasmid DNAs encoding mStayGold (#212020; pcDNA3/mStayGold (c4) = UtrCH) or StayGold/E138D (#211363; pcDNA3–10His-mStayGold (E138D)), were purchased from Addgene. These plasmids were used as templates in the polymerase chain reaction (PCR) to amplify the DNA sequence of mStayGold and StayGold/E138D. The Phusion^®^ High-Fidelity PCR Master Mix with GC Buffer (Thermo Fisher Scientific), a 2× master mix consisting of Phusion Hot Start II DNA Polymerase, deoxynucleotides and optimized reaction buffer with MgCl_2_, particularly useful for sequences with high GC content, was used to ensure accurate DNA replication. PCR products were purified using the QIAquick PCR purification kit (QIAGEN).

mStayGold for C1 vector

Forward primer: 5’-ATCCACCGGTCGCCACCATGGTGTCTACAGGC-3’.

Reverse primer: 5’-GAGCTCGAGATCTGAGTCCGGACAGGTGGGCCTCCAG-3’.

StayGold/E138D.

Forward primer: 5’-ATCCACCGGTCGCCACCATGGCCAGCACGCCG-3’.

Reverse primer: 5’-GAGCTCGAGATCTGAGTCCGGAAAGATGAGCTTCTAA-3’.

mStayGold for N1 vector.

Forward primer: 5′- ATCCACCGGTCGCCACCATGGTGTCTACAGGC-3’.

Reverse primer: 5’-GTCGCGGCCGCTTTACAGGTGGGCCTCCAG-3’.

Tandem dimer of mStayGold.

Forward primer: 5’-GACGGTACCATGGTGTCTACAGGCG-3’.

Reverse primer: 5’-GCGACCGGTGGCAGGTGGGCCTCCAG-3′.

To swap the DNA sequences encoding eGFP in the peGFP-C1 and the peGFP-GR-C1 plasmids with the sequences for mStayGold or StayGold/E138D, the PCR-amplified sequences for mStayGold and StayGold/E138D and the peGFP-C1 plasmid encoding eGFP in the C1 vector or the peGFP-GR-C1 plasmid encoding eGFP-tagged full-length human glucocorticoid receptor α (GR) in the C1 vector [51], were digested using the restriction enzymes *Age*I-HF (New England Biolabs (NEB)) and *Xho*I (NEB) in the rCutSmart buffer (NEB) at 37 °C for 1 h. To minimize annealing of DNA fragments, the plasmid DNAs were treated with Quick CIP (NEB), followed by the purification using QIAquick nucleotide removal kit (QIAGEN). The PCR-amplified sequences of mStayGold or StayGold/E138D were inserted into the plasmid backbone DNAs (C1 vector or GR-C1 vector) and ligated using the Instant Sticky-end Ligase Master Mix (NEB). The resulting constructs were verified using DNA sequencing to ascertain that pmStayGold-C1 and pStayGold/E138D-C1 contain mStayGold and StayGold/E138D, respectively in the C1 vector, and that pmStayGold-GR-C1 is encoding both mStayGold and GR in the C1 vector.

To construct the tandem dimer of mStayGold, eGFP in the peGFP-N1 plasmid was swapped with mStayGold using the procedure with *Age*I-HF (NEB) and *Not*I (NEB). Another mStayGold was inserted into the pmStayGold-N1 with *Kpn*I-HF (NEB) and AgeI-HF (NEB).

### 2.3. Cell culture

HEK293 cells (American Type Culture Collection) were maintained in a humidified atmosphere containing 5 % CO_2_ at 37 °C in Dulbecco’s Modified Eagle Medium (DMEM; Gibco) supplemented with 10 % fetal bovine serum (Gibco) and 1 % penicillin-streptomycin (100 U/ml of penicillin and 100 μg/ml of streptomycin; Gibco). For FCS measurements, the cells were seeded in Nunc Lab-Tek 8-well Chambered Coverglass (Thermo Fisher Scientific) at the density of 2.0 × 10^4^ cells/well in 400 μl of cell culture medium.

24 h after seeding the cells in the 8-well chambered coverglass, the cells were transfected with 100 ng of plasmid DNAs encoding: eGFP, mStayGold, tandem dimer of eGFP [52] or mStayGold, StayGold/E138D, eGFP-GR or mStayGold-GR, using 0.2 μl/well of Lipofectamin 2000 (Thermo Fisher Scientific). Following a 24 h’ transfection in a humidified atmosphere containing 5 % CO_2_ at 37 °C, the cell culture medium was replaced with the phenol red-free medium FluoroBrite DMEM (Gibco) for live-cell fluorescence microscopy imaging and FCS.

For GR activation, the 2 mM Dex stock solution was thawed at room temperature, diluted to 500 nM using the phenol red-free FluoroBrite DMEM (Gibco) medium and the cell culture medium was replaced. Unless otherwise stated, Dex treatment lasted 30 min at 37 °C and 5 % CO_2_ in a humidified atmosphere. In control experiments, sham treatment with the cell culture medium FluoroBrite DMEM (Gibco) was used.

### 2.4. Confocal laser scanning microscopy and fluorescence correlation spectroscopy

Confocal Laser scanning microscopy (CLSM) imaging and FCS measurements were performed at 25 °C using an LSM880 (Carl Zeiss) microscope system equipped with a 405 nm laser, 488 nm Ar-ion laser, and a water immersion objective lens (C-Apochromat, 40×, 1.2 N.A., Corr, Carl Zeiss), a gallium arsenide phosphide (GaAsP) detector and photomultiplier tube (PMT) detectors. eGFP, mStayGold or StayGold/E138D were excited using the 488 nm laser line of the Ar-ion laser. The pinhole size was adjusted to 1 Airy unit (34 μm). For fluorescence detection, the GaAsP detector was used, and the wavelength range of 500–530 nm was collected. FCS measurements were performed in a series of 10 consecutive recordings, each individual recording lasting 20 s. For fluorophore brightness analysis as a function of excitation laser intensity, the following values were tested: 0.07, 0.11, 0.15, 0.21, 0.26, 0.38, 0.72 and 1.45 μW at the objective lens, corresponding to: 0.05, 0.09, 0.11, 0.15, 0.2, 0.29, 0.54 and 1.1 kW·cm^−2^. For glucocorticoid receptor measurements in live cells, laser intensity of 0.72 μW (0.54 kW·cm^−2^) was used.

For transfection efficiency analysis, transfected cells were stained using the cell-permeable nuclear dye NucBlue^™^ Live ReadyProbes^™^ Reagent (Hoechst33342; Invitrogen). Hoechst33342 and eGFP, mStayGold or StayGold/E138D were excited using the 405 nm laser and the 488 nm laser line of the Ar-ion laser, respectively. The pinhole size was set to a maximum value (600 μm) to observe all fluorescence. Hoechst33342 fluorescence was detected in the 419–500 nm range using a PMT detector, and in the 500–550 nm range for eGFP, mStayGold or StayGold/E138D using the GaAsP detector. To minimize the signal crosstalk, the multi-track imaging mode was used.

### 2.5. FCS data analysis

FCS data were analyzed using the ZEN software (Carl Zeiss). Autocorrelation curves (ACCs) were generated by plotting as a function of the lag time (τ) the autocorrelation function (G(τ)) calculated as:

(1) $$G(\tau )=\frac{\left\langle I(t)\;∙\;I(t\;+\;\tau )\right\rangle }{{\left\langle I(t)\right\rangle }^{2}}$$

where I(t) is the fluorescence intensity at time t.

The autocorrelation curves were analyzed by fitting, using for eGFP, mStayGold or StayGold/E138D in live cells a one-component anomalous diffusion model [21]:

(2) $$G(\tau )=G(\infty )\;+\;\left(1\;+\;\frac{F_{t}\;∙\;e^{-\frac{\tau }{\tau_{t}}}}{1\;-\;F_{t}}\right)\;∙\;\frac{1}{N}\;∙\;\left[\frac{1}{1\;+\;{\left(\frac{\tau }{\tau_{D}}\right)}^{\alpha }}\right]\;∙\;\left[\frac{1}{1\;+\;\frac{1}{S^{2}}\;∙\;{\left(\frac{\tau }{\tau_{D}}\right)}^{\alpha }}\right]\frac{1}{2}$$

A two-component free 3D diffusion model was used for eGFP-GR and mStayGold-GR in the cytoplasm in untreated live cells (Eq. (3) with i=2), whereas a three-component free 3D diffusion model (Eq. (3) with i=3) was used for eGFP-GR or mStayGold-GR in the nucleus after treatment with 500 nM Dex:

(3) $$G(\tau )=G(\infty )\;+\;\left(1\;+\;\frac{F_{t}\;∙\;e^{\;-\;\frac{\tau }{\tau_{t}}}}{1\;-\;F_{t}}\right)\;∙\;\frac{1}{N}\;∙\;\sum_{i=1}^{2\;\mathrm{or}\;3}F_{i}\;∙\;\left(\frac{1}{1\;+\;\frac{\tau }{\tau_{D,i}}}\right)\cdot {\left(\frac{1}{1\;+\;\frac{\tau }{S^{2}∙\tau_{D,i}}}\right)}^{\frac{1}{2}}$$

In Eq. (2) and Eq. (3), $F_{t}$ is the average fraction of fluorescent molecules in the triplet state; $\tau_{t}$ is the average relaxation time of the triplet state; N is the average number of fluorescent molecules in the effective volume element; $F_{i}$ is the relative molar fraction of the i-th component ($F_{1}\;+\;F_{2}=1$ in the two-component free 3D diffusion model and $F_{1}\;+\;F_{2}\;+\;F_{3}=1$ in three-component free 3D diffusion model); $\tau_{D,i}$ is the average diffusion time of the i-th component; S is the structure parameter (S=z/w) which is the ratio of the axial (z) to lateral (w) radii of the effective volume element; G(∞) is the offset at infinite lag time. In Eq. (2), α is the anomalous diffusion exponent that is α≠1 for translational motion through complex/disordered systems and α=1 for normal, Brownian motion.

Since GRs bind in the cell nucleus to the genomic DNA, we have also performed fitting analysis using the so-called binding models (for details, see Supplementary Information), a two-component free 3D diffusion with one-component binding (Eq. (4)) and the one-component free 3D diffusion with two-component binding (Eq. (5)):

(4) $$G(\tau )=G(\infty )\;+\;\left(1\;+\;\frac{F_{t}\;∙\;e^{-\frac{\tau }{\tau_{t}}}}{1\;-\;F_{t}}\right)\;∙\;\frac{1}{N}\;∙\;\left[F_{1}\;∙\;\left(\frac{1}{1\;+\;\frac{\tau }{\tau_{D,1}}}\right)\;∙\;\left(\frac{1}{1\;+\;\frac{\tau }{S^{2}•\tau_{D,1}}}\right)\frac{1}{2}\right.\;\left.\;+\;F_{2}\;∙\;\left(\frac{1}{1\;+\;\frac{\tau }{\tau_{D,2}}}\right)\;∙\;\left(\frac{1}{1\;+\;\frac{\tau }{S^{2}∙\tau_{D,2}}}\right)\frac{1}{2}\;+\;\left(1\;-\;F_{1}\;-\;F_{2}\right)\;∙\;e^{\left(\;-\;k_{B,1}∙\tau \right)}\right]$$

(5) $$\begin{array}{cc} G(\tau )= & G(\infty )\;+\;\left(1\;+\;\frac{F_{t}\;∙\;e^{-\frac{\tau }{\tau_{t}}}}{1\;-\;F_{t}}\right)\;∙\;\frac{1}{N}\;∙\;\left[F_{1}\;∙\;\left(\frac{1}{1\;+\;\frac{\tau }{\tau_{D,1}}}\right)\;∙\;\left(\frac{1}{1\;+\;\frac{\tau }{S^{2}∙\tau_{D,1}}}\right)\frac{1}{2}\right.\\ \; & \left.\;+\;F_{2}\;∙\;e^{\left(\;-\;k_{B,1}\;∙\;\tau \right)}\;+\;\left(1\;-\;F_{1}\;-\;F_{2}\right)∙e^{\left(\;-\;k_{B,2}∙\tau \right)}\right] \end{array}$$

where $k_{B,1}$ and $k_{B,2}$ are rate constants determining the kinetics of GR binding to the genomic DNA [53].

When fitting Eq. (4) or Eq. (5) to the experimental ACCs, diffusion time of the first component $\left(\tau_{D,1}\right)$ was fixed to the value measured for eGFP-GR or mStayGold-GR in the cytoplasm before Dex treatment, since this component originates from the free 3D diffusion of these molecules in the cytoplasm/nucleus.

Values of the lateral (w) and axial (z) radii of the effective volume element were determined in calibration measurements using the ATTO488 dye (DATTO488 = 400 μm^2^s^−1^) [54]:

(6) $$w=\sqrt{4\;∙\;D_{\mathrm{ATTO488}}\;∙\;\tau_{\mathrm{D,ATTO488}}}$$

(7) z=w∙S

(8) $$V_{\mathrm{eff}}=\pi^{\frac{3}{2}}\;∙\;w^{2}\;∙\;z$$

where $D_{\mathrm{ATTO488}}$ is the diffusion coefficient of ATTO488, and $\tau_{D,ATTO488}$ is the diffusion time of ATTO488. $V_{\mathrm{eff}}$ is the effective detection volume. The diffusion coefficients were calculated from the corresponding experimentally measured diffusion times and the lateral radius determined as described above (Eq. (6)).

The average number of eGFP, mStayGold, StayGold/E138D, eGFP-GR and mStayGold-GR were obtained from the fitting analysis. To compare the apparent brightnesses of eGFP, mStayGold and StayGold/E138D and quantitatively characterize homodimerization of eGFP-GR and mStayGold-GR, counts *per* second *per* molecule (*CPM*) were calculated as:

(9) $$CPM_{i}=\frac{I_{i}}{N_{i}}$$

where i denotes eGFP, mStayGold, StayGold/E138D, eGFP-GR or mStayGold-GR; $I_{i}$ is the average fluorescence intensity in counts *per* second, and $N_{i}$ the number of molecules of the corresponding species determined by fitting analysis.

We have furthermore calculated the fraction of monomer ($F_{m}$) and homodimer ($F_{d}$) from the relative molecular brightness (R) as follows [55]:

(10) $$R=\frac{CPM_{j}}{CPM_{k}}$$

(11) $$F_{m}=\frac{4\;-\;2R}{3\;-\;R}$$

(12) $$F_{d}=\frac{R\;-\;1}{3\;-\;R}$$

where j denotes eGFP-GR or mStayGold-GR; k denotes eGFP or mStayGold; *CPM* is the counts *per* second *per* molecule calculated using (Eq. (9)). We confirmed that the brightness of homodimers is twice that of a monomer, $CPM_{\mathrm{eGFP}\;\mathrm{dimer}}^{\mathrm{norm}}=1.98\pm 0.08$; and $CPM_{\mathrm{mStayGold}\;\mathrm{dimer}}^{\mathrm{norm}}:1.97\pm 0.15$ (Fig. S1). The dissociation constant of GR homodimerization is determined by using both linear regression analysis without and with Deming method (Eq. (13)) and histogram analysis of the dissociation constant of GR homodimerization in each single-cell data:

(13) $$K_{d,\mathrm{homo}}=\frac{{\left[C_{m}\right]}^{2}}{\left[C_{d}\right]}$$

(14) $$\left[C_{m}\right]=F_{m}\;∙\;\left[C_{l}\right]$$

(15) $$\left[C_{d}\right]=F_{d}\;∙\;\left[C_{l}\right]$$

where $K_{\mathrm{d,homo}}$ denotes the dissociation constant of eGFP-GR or mStayGold-GR homodimers; [$C_{m}$] and [$C_{d}$] are the concentration of monomeric and homodimeric eGFP-GR or mStayGold-GR, respectively; [$C_{l}$] is the concentration of eGFP-GR or mStayGold-GR obtained from FCS measurements (with l denoting eGFP-GR or mStayGold-GR).

### 2.6. Massively parallel FCS integrated with fluorescence lifetime imaging microscopy (mpFCS/FLIM)

The optical setup for mpFCS/FLIM is described in detail in our previous work [21]. Briefly, the instrument was built using an inverted epi-fluorescence microscope Axio Observer D1 equipped with a C-Apochromat 63×/1.2 W Corr objective lens (Carl Zeiss, Germany) and a filter set (Filter Set 38 HE; Ex BP 470/40 nm, long pass dichoric mirror with a cutoff wavelength of 495 nm, Em BP 525/50 nm) for eGFP, mStayGold and StayGold/E138D. A direct current-modulated picosecond 482 nm laser (Laser Diode Head LDH-D-C-485, PicoQuant, Germany) was used as the excitation light source. The laser was driven by a Picosecond Pulsed Diode Laser Driver (PDL 800-D), allowing operation in either continuous wave (CW) mode or pulsed mode. A Diffractive Optical Element (DOE; Holoeye, Germany) was used to precisely create a spot-wise 16 × 16 illumination pattern that matches centrally positioned 16 × 16 single-photon avalanche diodes (SPADs) on the SPC3 camera with 64 × 32 circular SPADs (Micro Photon Devices MPD, Italy). To enable fast sample localization, an 18.0 megapixel digital single-lens reflex (DSLR) camera EOS 600D (pixel size of 18.5 μm^2^ and pixel pitch of 4.3 μm; Canon Inc., Japan) was coupled to the side port of the microscope opposite to the SPC3 camera, and the light path between the two camera ports was manually switched.

### 2.7. mpFCS/FLIM data acquisition and analysis

For mpFCS measurements, 1,048,575 frames were acquired at a temporal resolution of 20 μs/frame. Fluorescent molecules were excited using 2.1 μW (1.43 kW·cm^−2^) laser power *per* spot (total 535 μW), yielding 256 fluorescence intensity fluctuation traces recorded over 20.97 s. For mStayGold-GR measurements, 15 min after treatment with 500 nM Dex, the same number of frames was acquired at a temporal resolution of 40 μs/frame, lasting 41.94 s.

The ACCs were calculated using our home-built dedicated mpFCS software for fast auto- and cross-correlation analysis by parallel signal processing using a graphic processing unit (GPU), allowing temporal autocorrelation across all pixels in the image frame in 1 s and cross-correlation between first- and second-order neighbor pixels in several seconds [21]. Fitting analysis using the one-component anomalous diffusion model (Eq. (2)), was performed for eGFP, StayGold/E138D and mStayGold. To this aim, the Origin Data Analysis and Graphing software (OriginLab) was used. Fitting analysis for mStayGold-GR was performed by two-component free diffusion model (Eq. (3), i=2) in the cytoplasm and the three-component free diffusion model (Eq. (3), i=3) in the nucleus.

For glucocorticoid receptor translocation analysis, we analyzed two-foci cross-correlation curves (tfCCCs) as previously described [22]. A schematic diagram on the tfCCC analysis is drawn in Fig. S2. In brief, the Origin Data Analysis and Graphing software (OriginLab) was used to compute the tfCCCs between foci in the cytoplasm and nucleus. To reduce the calculation cost, the photon count data was integrated into 4 ms bins. Of note, the photobleaching correction before tfCCCs calculation was not needed in the case of mStayGold due to its remarkable photostability!

The arbitrarily assigned as forward $G_{c}^{c,n}(\tau )$, reflecting molecular transport from the cytoplasm to the cell nucleus, and the backward $G_{c}^{n,c}(\tau )$, designating the cell nucleus to cytoplasm translocation tfCCCs are defined as follows:

(16) $$G_{c}^{c,n}(\tau )=\frac{\left\langle I^{n}(t)\;∙\;I^{c}(t\;+\;\tau )\right\rangle }{\left\langle I^{n}(t)\right\rangle \;∙\;\left\langle I^{c}(t)\right\rangle }$$

(17) $$G_{c}^{n,c}(\tau )=\frac{\left\langle I^{c}(t)\;∙\;I^{n}(t\;+\;\tau )\right\rangle }{\left\langle I^{n}(t)\right\rangle \;∙\;\left\langle I^{c}(t)\right\rangle }$$

where $I^{n}(t)$ and $I^{c}(t)$ are time series of fluorescence intensity fluctuations recorded in the selected pixels in the nucleus and cytoplasm, respectively.

We further calculate the so-called subtracted tfCCC, $G_{c,\mathrm{sub}}(\tau )$:

(18) $$G_{c,sub}(\tau )=G_{c}^{c,n}(\tau )\;-\;G_{c}^{n,c}(\tau )$$

to readily visualize the translocation direction as positive (nuclear import) or negative peaks (nuclear export).

FLIM data acquisition and analysis were performed as previously described [21,56]. In brief, the signal was acquired for 5 min to obtain low-noise FLIM curves necessary to enable precise fitting analysis. By fitting a one-component exponential decay function:

(19) $$I(t)=I_{off}\;+\;Aexp\left(\;-\;\frac{t\;-\;t_{0}}{\tau_{f}}\right)$$

to the FLIM curves using our dedicated mpFCS software, fluorescence lifetime of eGFP, StayGold/E138D and mStayGold in live-HEK cells were determined. In Eq. (19), I(t) denotes photon cunts at time t, $I_{\mathrm{off}}$ is the offset due to background photon counts, A is the amplitude of the FLIM curve, and $\tau_{f}$ is the fluorescence lifetime of eGFP, StayGold/E138D or mStayGold. In each cell, the fluorescence lifetime was calculated as an average from 16 to 100 independent positions in the cell.

## 3. Results

### 3.1. Monomeric StayGold variants are twice as bright as eGFP in live HEK cells

**Fig. 1.**
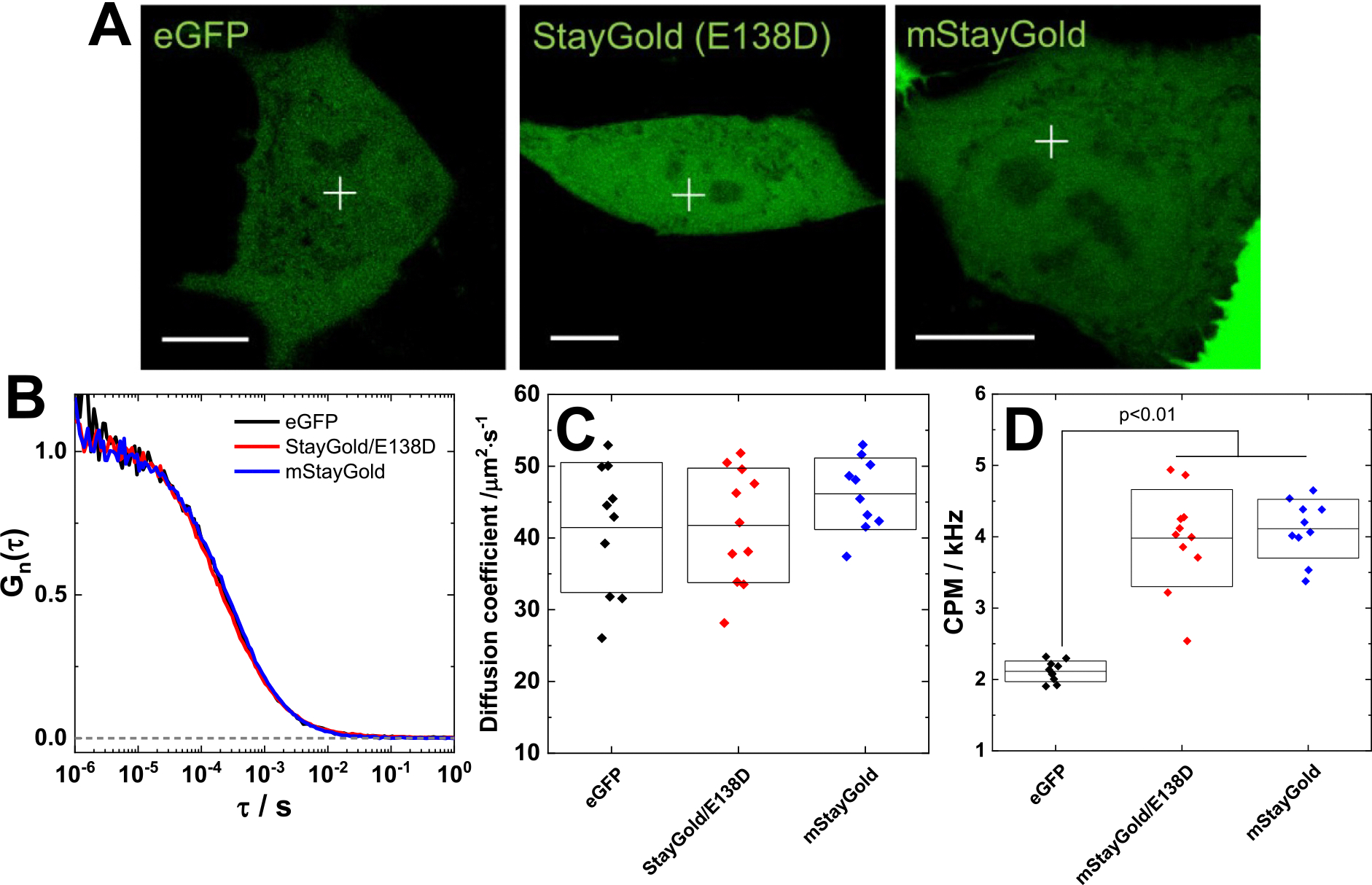
The intracellular distribution and diffusion of monomeric StayGold variants are indistinguishable from those of eGFP, but they are twice as bright. **(A)** Confocal Laser Scanning Microscopy (CLSM) images of HEK cells expressing eGFP (left), StayGold/E138D (middle) or mStayGold (right). White cross indicates the position at which FCS measurement were performed. Scale bar: 10 μm. **(B)** Autocorrelation curves (ACCs) normalized to the same amplitude, $G_{n}(\tau )=1$ at τ=10μs, reflecting the dynamics of eGFP (black), StayGold/E138D (red) and mStayGold (blue) in HEK cells. **(C)** Diffusion coefficients of eGFP (black), StayGold/E138D (red) and mStayGold (blue) derived by fitting analysis. **(D)** Apparent molecular brightness of eGFP (black), StayGold/E138D (red) and mStayGold (blue) expressed as counts *per* second *per* molecule (CPM). The middle lines and box edges indicate the mean value and standard deviation, respectively. Data analysis was performed using the one-way ANOVA test with *post hoc* Tukey test. A statistically significant difference was not observed, *p* > 0.05.

To measure the brightness of the different GFP variants in live cells, we transiently expressed eGFP, mStayGold or StayGold/E138D in live HEK cells (Fig. 1A) and used conventional spFCS to measure their brightness (Fig. 1B–D). Our unique mpFCS/FLIM system was used to measure fluorescence lifetime (Fig. S3).

Temporal ACCs in live cells (Fig. 1B), reflecting the diffusion of the investigated GFP variants, overlapped to a very large extent, suggesting that their diffusion is similar. Fitting the one-component anomalous diffusion model to these ACCs (Fig. S4), diffusion times and the average number of molecules were determined, and the diffusion coefficients (Fig. 1C) and CPMs, reflecting on apparent molecular brightness (Fig. 1D) were calculated. As expected, diffusion coefficients and anomalous diffusion exponents were not significantly different between different GFP variants (Fig. 1C, S5A), whereas molecular brightness of both monomeric StayGold variants was twice that of eGFP (Fig. 1D). This may lead to the reduction of chi-square value *per* degree of freedom in the fitting analysis (Fig. S5C).

**Fig. 2.**
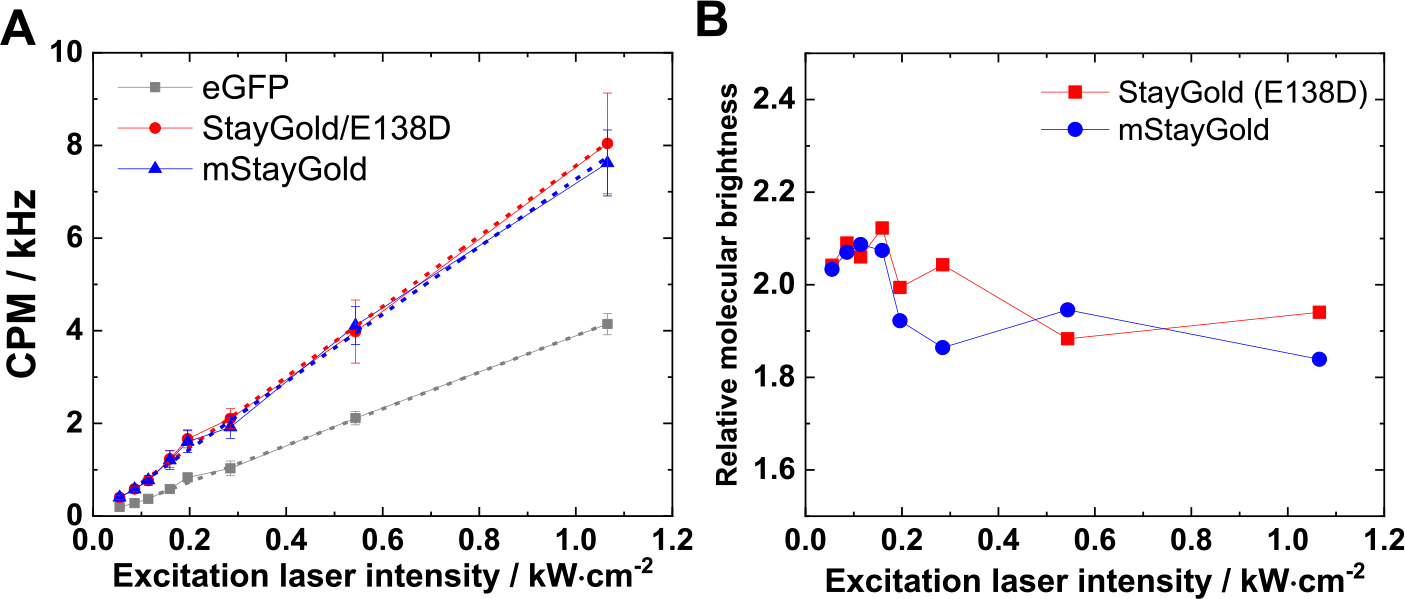
Excitation-intensity-independent brightness of monomeric StayGold variants compared to eGFP. **(A)** Apparent brightness of eGFP (black), StayGold/E138D (red) or mStayGold (blue) linearly increases as a function of the excitation laser intensity at 488 nm in the range of intensities tested. Average CPM values and corresponding standard deviations were calculated from 10 single-cell data recordings. Linear regression analysis yielded Pearson’s correlation coefficient > 0.99. The slopes, reflecting the average apparent molecular brightness across the range of laser intensities tested, were: $slope_{I}^{\mathrm{eGFP}}=2.95\pm 0.04$, ${slope}_{I}^{\mathrm{StayGold}/E\mathrm{138}D}$ 5.67 ± 0.09 and $slope_{I}^{mStayGold}=5.42\pm 0.11$. **(B)** Relative molecular brightness as a function of excitation intensity. In the range of excitation laser intensities tested, $R_{\mathrm{StayGold}/E138D}=2.02\pm 0.08$ and $R_{\mathrm{mStayGold}}=1.98\pm 0.1$. The two-tailed Student’s t-test indicated no significant difference between the monomeric StayGold variants.

Apparent molecular brightness measured for all three GFP variants shows linear correlation with the excitation laser intensity at the objective lens (Fig. 2A). Apparent molecular brightness of mStayGold and StayGold/E138D relative to that of eGFP, *i.e.*, relative molecular brightness according to Eq. (10), is R=(2±0.07), and remains roughly the same in the whole range of laser intensities tested (Fig. 2B), suggesting that mStayGold and StayGold/E138D achieve the same signal-to-noise ratio with half the laser power. Of note, while a downward trend in relative molecular brightness as a function of excitation intensity is apparent in Fig. 2B, the difference is not statistically significant in the range of excitation intensities tested. However, if confirmed for even higher excitation intensities, this downward trend may suggest that subtle differences in saturation kinetics may exist between the monomeric StayGold variants and eGFP.

Fluorescence lifetime (Fig. S3) of both monomeric StayGold variants, $\tau_{f}^{m\mathrm{StayGold}}=\left(2.90\pm 0.01\right)\;ns$ and $\tau_{f}^{\mathrm{StayGold}/E138D}=\left(2.88\pm 0.02\right)\;ns$, is somewhat longer than that of eGFP, $\tau_{f}^{eGFP}=\left(2.50\pm 0.03\right)\;ns$, in good agreement with previous report [43].

### 3.2. Enhanced photostability of monomeric StayGold variants enables photobleaching-free mpFCS measurements

**Fig. 3.**
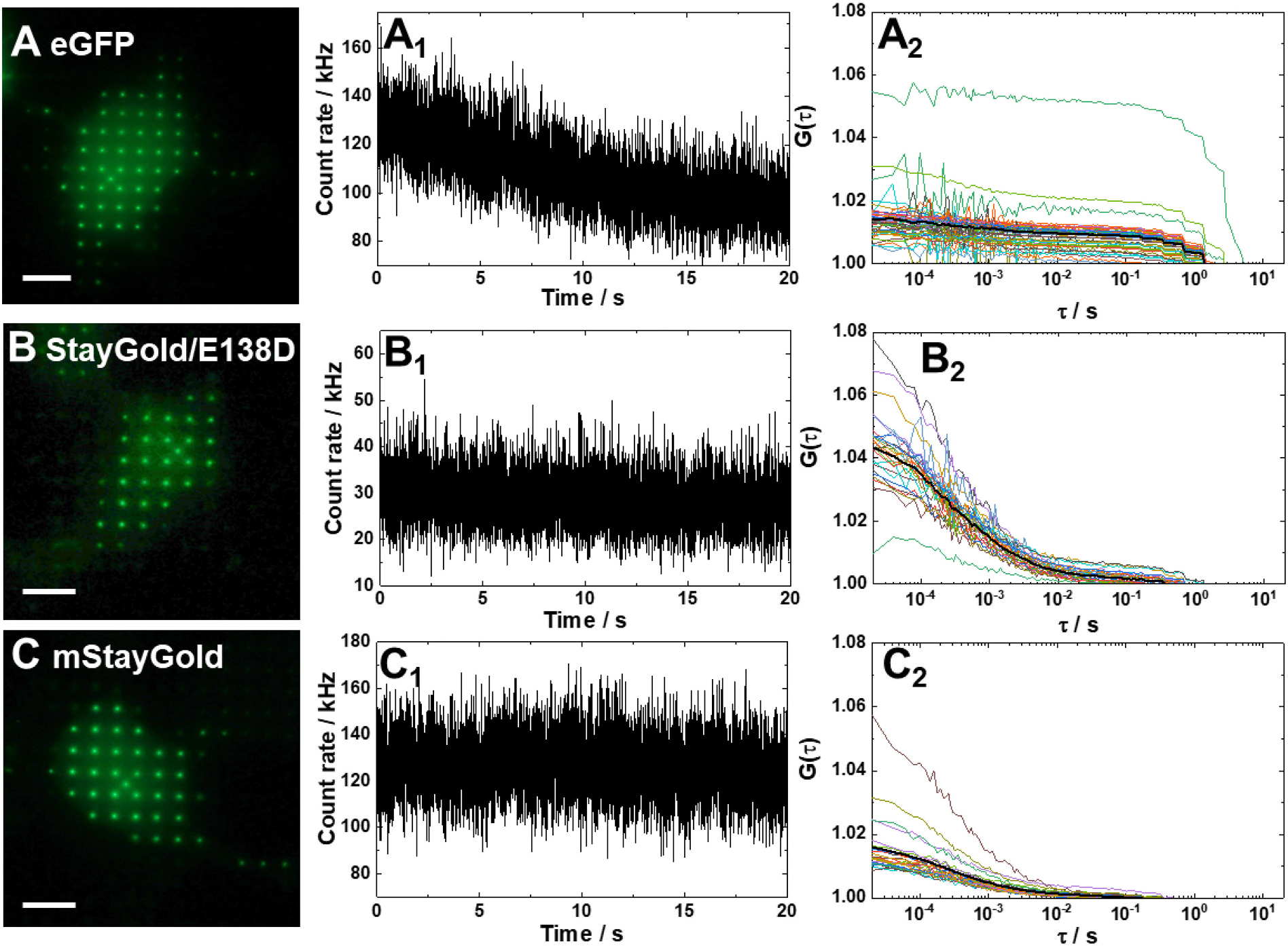
Monomeric StayGold variants with enhanced photostability facilitate photobleaching-free mpFCS measurements in live cells. **(A, B, C)** Fluorescence microscopy images showing the intracellular localization of eGFP (A), StayGold/E138D (B) and mStayGold (C) in live HEK cells acquired using spot-wise illumination and the DSLR Canon camera coupled to the side port of the mpFCS system. Scale bar: 10 μm. **(A**_**1**_**, B**_**1**_**, C**_**1**_**)** Corresponding fluorescence intensity fluctuation time series showing prominent photobleaching in eGFP-expressing HEK cells (A_1_) and photobleaching-free time series acquired in mStayGold- or StayGold/E138D-expressing cells (B_1_ and C_1_). **(A**_**2**_**, B**_**2**_**, C**_**2**_**)** Corresponding temporal autocorrelation curves (ACCs) derived in all cells from first acquired time series in each cell. Autocorrelation curves of eGFP after the photobleaching are shown in Fig. S7A.

Results of mpFCS measurements in live HEK cells transiently expressing eGFP, mStayGold or StayGold/E138D are summarized in Fig. 3. In line with data shown in Fig. 1A, mpFCS shows uniform fluorescence intensity distribution of all three GFP variants in live HEK cells (Fig. 3A–C). However, fluorescence intensity significantly decreased over time for mpFCS measurements in HEK cells expressing eGFP (Fig. 3 A_1_), while it remained unchanged in HEK cells expressing StayGold/E138D (Fig. 3 B_1_) or mStayGold (Fig. 3 C_1_). This was also reflected in the raw ACCs, derived without applying the photobleaching correction (Fig. 3 A_2_–C_2_). Here, temporal autocorrelation analysis yielded for measurements in eGFP-expressing cells ACCs with two characteristic decay times and a high relative amplitude of the component with the long decay time that is characteristic of photobleaching (Fig. 3 A_2_ and S6A). This is markedly different from the ACCs acquired in StayGold/E138D- or mStayGold-expressing cells (Fig. 3 B_2_ and Fig. 3 C_2_, respectively) that showed a high relative amplitude of the component with the short decay time, which is reflecting protein diffusion within the cells, and a very low relative amplitude, if at all, for the component with the long decay time that is characteristic of drawn-out fluorescence intensity fluctuation from slow processes (Fig. S6B). Photobleaching correction in the fluorescence intensity fluctuation using the 4th-degree polynomial function to account for slow changes in the average fluorescence intensity due to photobleaching, makes it possible to remove these slower decays (Fig. S6C, D).

Of note, following several initial mpFCS measurements in eGFP-expressing cells a more stable fluorescence signal is obtained, yielding ACCs with a short decay time component and a barely visible long decay time component that is characteristic of photobleaching (Fig. S7A). However, the corresponding single-pixel ACCs were noisier than those acquired for monomeric StayGold variants (Fig. S7B). And, the low SNR affected the precision of the curve fitting analysis (Fig. S5C), causing large deviations in the calculated diffusion coefficients and anomalous diffusion exponents (Fig. S5B and S7C).

In addition, eGFP apparent molecular brightness, reflected through CPM, seems to be 4 times lower than that of mStayGold or StayGold/E138D (Fig. S7D). This is possibly due to dynamic quenching of eGFP, presumably by reactive oxygen species (ROS) generated during pre-bleaching (Fig. S8).

### 3.3. Enhanced brightness and photostability of mStayGold improve live-cell analysis of glucocorticoid receptor (GR) binding to genomic DNA

**Fig. 4.**
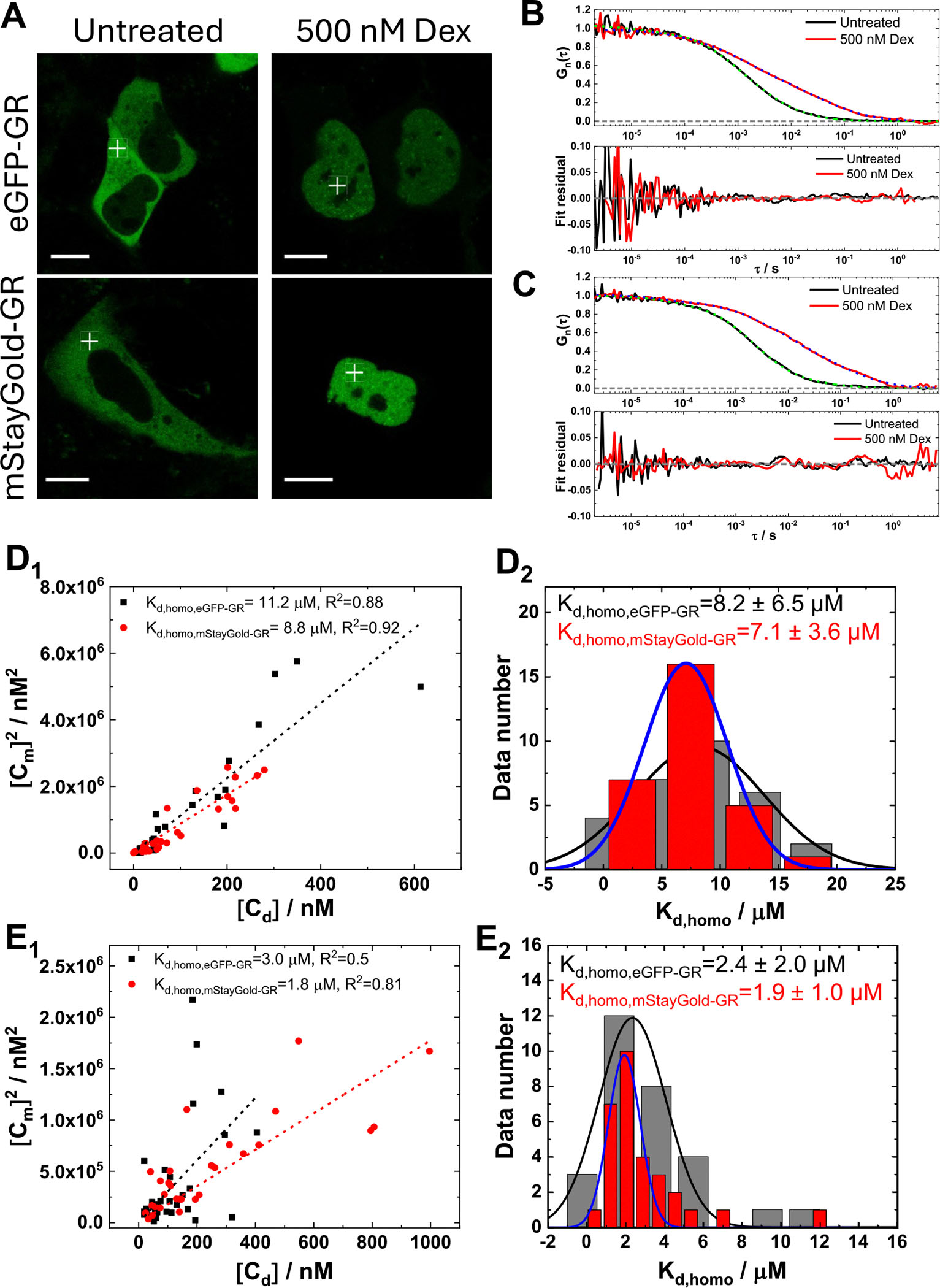
Enhanced brightness and photostability of mStayGold improve live-cell analysis of glucocorticoid receptor (GR) homodimerization by brightness analysis. **(A)** Confocal fluorescence microscopy images of HEK cells expressing eGFP-GR (top) or mStayGold-GR (bottom) in untreated cells (left) or cells treated with 500 nM Dex (right). White crosses indicate the positions at which FCS measurements were performed. Scale bar: 10 μm. **(B, C)** Autocorrelation curves (ACCs) normalized to the same amplitude, $G_{n}(\tau )=1$ at τ=10μs, (top) and corresponding fit residuals (bottom), reflecting the dynamics of eGFP-GR (B) and mStayGold-GR (C) in the cytoplasm of untreated HEK cells (black) and the nucleus in cells treated with 500 nM Dex for 30 min (red). Green- and blue-dashed curves represent the best fit of two-component and three-component free diffusion model, respectively. **(D**_**1**_**, D**_**2**_**, E**_**1**_**, E**_**2**_**)** Apparent dissociation constant of eGFP-GR homodimers (black) and mStayGold-GR homodimers (red) in the cytoplasm of untreated cells (D_1_, D_2_) and in the nucleus of cells treated with 500 nM Dex for 30 min (E_1_, E_2_). Linear regression analysis (D_1_, E_1_) and Gaussian curve fitting applied to histograms (D_2_, E_2_) determined the dissociation constant, $K_{d,\mathrm{homo}}$, for the eGFP-GR and the mStayGold-GR homodimer complexes.

Functional proteins with lower mobility such as DNA-bound transcription factors are more easily photobleached than their unbound counterparts that freely diffuse through the nuclear lumen. Using the glucocorticoid receptor (GR) as a transcription factor model, we have characterized in live HEK cells mStayGold-GR binding (Fig. 4) – of note, due to lower transfection efficiency of StayGold/E138D in HEK cells (Fig. S9), we have not proceeded with StayGold/E138D-GR.

CLSM imaging clearly showed the cytoplasmic localization of both GR constructs, eGFP-GR and mStayGold-GR, in untreated HEK cells (Fig. 4A, left). After 30 min treatment with 500 nM Dex, nuclear localization is clearly observed (Fig. 4A, right). FCS measurements reveal slower diffusion in the cell nucleus of both GR constructs, due to GR binding to the genomic DNA (Fig. 4B and C). To characterize GR diffusion properties, we tested several fitting models for both, eGFP-GR and mStayGold-GR in the cytoplasm of untreated cells (Fig. S10A1 and B_1_) and in the cell nucleus of Dex-treated cells (Fig. S10A_2_, A_3_, B_2_ and B_3_). In untreated cells, both one-component anomalous diffusion model and two-component free 3D diffusion model could well fit the ACCs. However, the fit residues were larger when in subsequent analyses the diffusion time obtained from the one-component anomalous diffusion model was used. We have therefore decided to apply throughout the two-component free diffusion model. This model is consistent with the notion that the cytoplasmic pool of GRs consists of freely diffusing GRs and GRs bound to other molecules, forming cytoplasmic components of lower mobility. In the nucleus of Dex-treated cells, three-component models (Fig. S10A_3_ and B_3_) were needed as the agreement with two-component models was poor (Fig. S10A_2_ and B_2_). Fitting residuals were, however, nearly identical between the free diffusion model (Eq. (3)) and the binding model (Eqs. (4) and (5)). We have therefore decided to proceed using the three-component free 3D diffusion model (Eq. (3)), fixing the diffusion time of the first component to the value determined for free 3D diffusion of GR in the cytoplasm. The second and the third component, related to GR binding to the genomic DNA, were allowed to freely vary. Superior mStayGold brightness and photostability lead to less noisy ACCs and significantly improved chi-squared values (Fig. S11).

The diffusion time of mStayGold-GR is longer than that of eGFP-GR, as evident from the shift of the ACC to longer lag times (Fig. S12A and B), yielding in the cytoplasm of untreated cells $\tau_{D}^{mStayGold\;-\;GR}=\left(1.8\pm 0.6\right)\;ms)$ and ($\tau_{D}^{eGFP\;-\;GR}=\left(1.0\pm 0.3\right)\;ms$ (Fig. S12C_1_). This apparent difference in diffusion time is likely because of eGFP photobleaching before it exits the effective volume element, due to which it appears as if the molecule has traversed it in a shorter time. Faster eGFP photobleaching compared to mStayGold may also explain why the fraction of bound mStayGold-GR appears to be higher than that of eGFP-GR (Fig. S12D_1_ and D_2_).

DNA-bound fraction did not show clear concentration dependence (Fig. S13), likely due to the narrow range of GR concentration tested.

### 3.4. Enhanced brightness and photostability of mStayGold improve the precision of determining the dissociation constant of glucocorticoid receptor homodimerization via molecular brightness analysis

Molecular brightness of fluorescently labelled proteins is sensitive to homo-oligomerization, making molecular brightness analysis a powerful tool for characterizing homo-oligomerization. Classical view on GR activation is that ligand binding to cytoplasmic GR dissociates the heteromolecular complex in which it resides, leading to GR translocation and subsequent homodimerization and possibly also homotetramerization in the cell nucleus, followed by the homo-GR complexes binding to GREs to regulate gene expressions. Given that GR homotetramers were only observed in genetically modified cells containing many GRE sequences on the mouse mammary tumor virus (MMTV) promoter array [47,57,58], and that even in these cells the GR homodimer in the nucleoplasm was significantly more abundant than the homotetramer [47,59], we have, in the first approximation, disregarded GR homotetramerization and used brightness analysis to examine GR homodimerization in the cytoplasm and cell nucleus.

As a first step, we measured the apparent brightness of eGFP and mStayGold in cells expressing these fluorescent proteins only and used these values to determine the relative molecular brightnesses (R) of eGFP-GR and mStayGold-GR against the apparent brightness of the corresponding monomeric GFP variant eGFP or mStayGold, respectively (Eq. (10)). We further calculated the monomeric/homodimeric fractions, $F_{m}$ and $F_{d}$, according to Eq. (11) and Eq. (12), respectively. Linear regression analysis without the Deming method of the relationship ${\left[C_{m}\right]}^{2}=f\left(C_{d}\right)$ according to Eq. (13), yielded the dissociation constant for GR homodimerization ($K_{d\mathrm{,homo}}$) (Fig. 4 D and E). In the cytoplasm of untreated HEK cells, the $K_{d\mathrm{,homo}}$ values were determined to be $K_{d,\mathrm{homo},\mathrm{cyto}}^{eGFP\;-\;GR}=11.2\;\mu M$ (R^2^_=_ 0.88) and $K_{d,\mathrm{homo,cyto}}^{m\mathrm{StayGold}\;-\;GR}=8.8\;\mu M$ (R^2^ = 0.92) (Fig. 4D1). Since each measurement enables us to compute the dissociation constant, histogram analysis was applied, yielding $K_{d,homo,cyto}^{eGFP\;-\;GR}=\left(8.2\pm 6.5\right)\;\mu M$ and $K_{d,homo,cyto}^{mStagGold\;-\;GR}=\left(7.1\pm 3.6\right)\mu M$ (Fig. 4 D2), which are in good agreement with the values obtained by linear regression analysis.

Following the same procedure, the dissociation constant of eGFP-GR or mStayGold-GR homodimer complexes in the nucleus of Dex-treated cells were determined to be $K_{d,homo,nuc}^{eGFP\;-\;GR}=3.0\;\mu M$ (R^2^ = 0.5) and $K_{d,\mathrm{homo,}\;\mathrm{nuc}}^{\mathrm{mStayGold}\;-\;GR}=1.8\;\mu M$ (R^2^ = 0.81) (Fig. 4 E1). Similar dissociation constant values were obtained by histogram analysis, yielding $K_{d,homo,nuc}^{eGFP\;-\;GR}=\left(2.4\pm 2.0\right)\;\mu M$ and $K_{d,\mathrm{homo},nuc}^{m\mathrm{StayGold}\;-\;GR}=\left(1.9\pm 1.0\right)\;\mu M$ (Fig. 4 E2). These values are in good agreement with data from live U2OS cells obtained using Fluorescence Cross-Correlation Spectroscopy (FCCS) [32]. We also tested linear regression analysis with the Deming method (Fig. S14), observing that fitting the scattered data of eGFP-GR in cells treated with 500 nM Dex, is not satisfactory. The Deming method required a dataset with small deviations to determine the dissociation constants, and the mStayGold-GR data met these requirements.

As expected, the relative standard deviation (defined as the ratio of the sample standard deviation to the sample average) was smaller for measurements using the brighter and more photostable mStayGold fluorophore than eGFP, as can be seen from the obtained values: 0.79 *vs* 0.51 in the cytoplasm of untreated cells and 0.83 *vs* 0.52 in the nucleus of Dex-treated cells. But, even in measurements where mStayGold was used, the relative standard deviation was rather high, around 0.5, suggesting that other factors such as heterogeneity of chromatin structure for measurements at different positions in the cell nucleus affect the dispersion of the data.

### 3.5. mStayGold facilitates spatial mapping of glucocorticoid receptor dynamics and transport

**Fig. 5.**
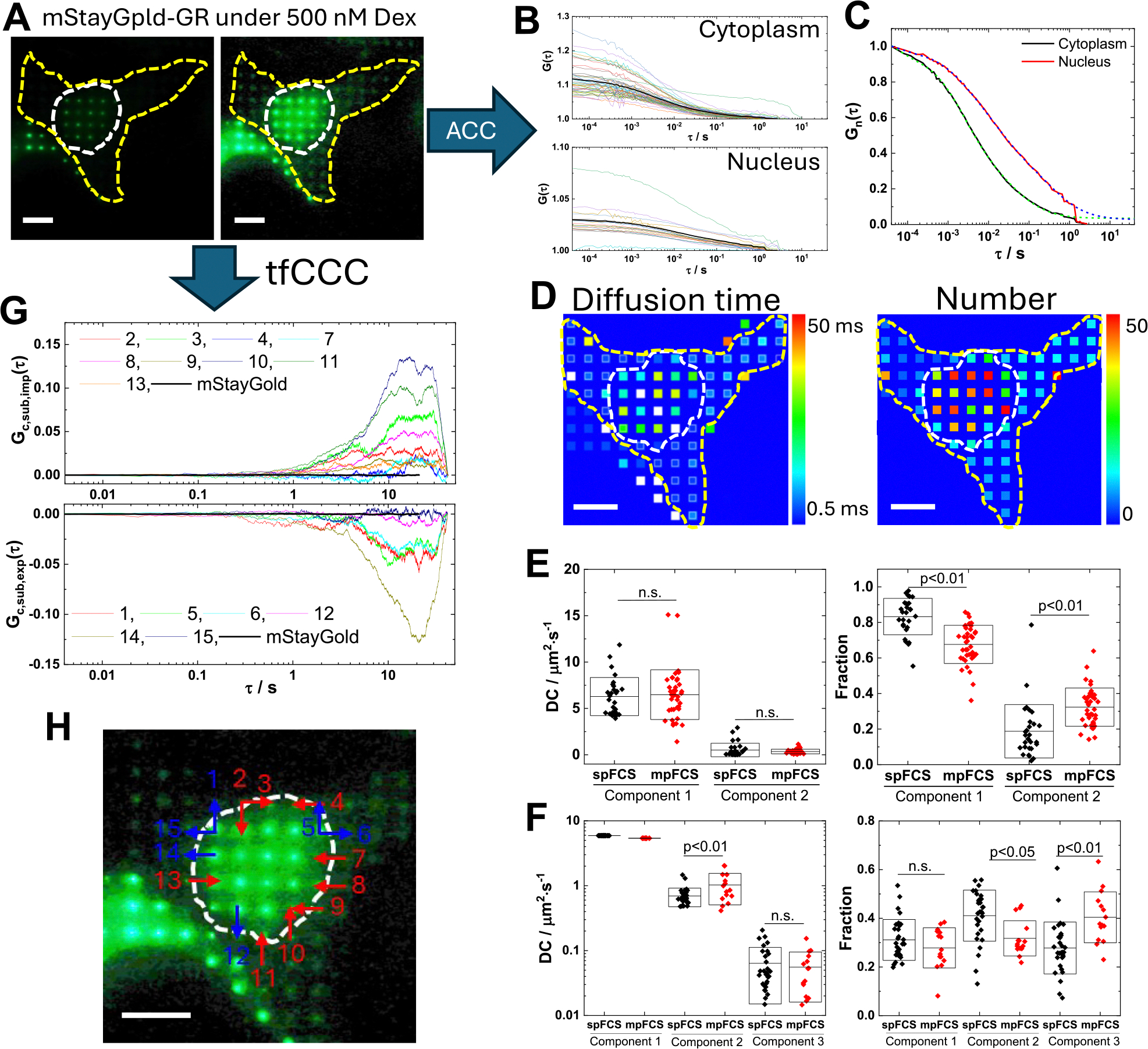
Enhanced brightness and photostability of mStayGold improve live-cell analysis of GR diffusion and nuclear import/export. **(A)** Fluorescence microscopy images showing mStayGold-GR distribution in a live HEK cell acquired after 15 min incubation with 500 nM Dex using spot-wise illumination and the DSLR Canon camera coupled to the side port of the mpFCS system. *Left image:* Original fluorescence image without any change in brightness and contrast. *Right image:* Image brightness was adjusted to enhance contrast and render the cytoplasmic component visible. The yellow and white dashed lines indicate the positions of the cytoplasm and the nucleus, respectively. Scale bar: 10 μm. **(B)** Autocorrelation curves (ACCs) recorded in the cytoplasm (top) and the cell nucleus (bottom). **(C)** Average ACCs recorded in the cytoplasm (black) and the cell nucleus (red) normalized to the same amplitude, $G_{n}(\tau )=1$ at τ=40μs. Green-dashed and blue-dashed lines represent the best fit of two-component or three-component free diffusion models, respectively. **(D)** Spatial mapping of mStayGold-GR diffusion time (left; image presented without applying any pixel masking) and average number of molecules (right; pixel masking outside the cell was applied to enhance visibility). Scale bar: 10 μm. **(E)** Comparison of diffusion coefficients (DCs) (left) and relative molar fractions (right) of components 1 and 2 in the cytoplasm of untreated cells acquired using spFCS (black) or cells treated with 500 nM Dex for 15 min acquired using mpFCS (red). **(F)** Comparison of diffusion coefficients (DCs) (left) and relative molar fractions (right) between components 1, 2 and 3 in the nucleus of cells treated with 500 nM Dex for 15 min acquired using spFCS (black) and mpFCS (red). Here, the first component was fixed in the fitting analysis using the first component measured in the cytoplasm as representative of free 3D diffusion of GR. In the boxplots (E and F), the sample mean and standard deviation are represented by the middle line and boxes, respectively. Statistical analysis was performed using the one-way ANOVA test with *post hoc* Tukey test. Lack of statistically significant difference is denoted as n.s., *p* > 0.05. **(G)** The so-called subtracted cross-correlation curves $G_{c,\mathrm{sub,imp}}(\tau )$ and $G_{c,\mathrm{sub,}\exp}(\tau )$ for all analyzed positions along the nuclear envelope shown in H. Briefly, two-foci cross-correlation analysis yields two two-foci cross-correlation curves (tfCCCs), one for each direction of calculation: from focus 1 in the cell nucleus to focus 2 in the cytoplasm, $G_{c,exp}(\tau )$, and conversely, from focus 2 in the cytoplasm to focus 1 in the cell nucleus, $G_{c,\mathrm{imp}}(\tau )$. Their difference, the so-called subtracted cross-correlation curve, $G_{c,\mathrm{sub}}(\tau )=G_{c,\mathrm{imp}}(\tau )\;-\;G_{c,\exp}(\tau )$, shows a positive amplitude for nuclear import, $G_{c,\mathrm{sub,imp}}(\tau )$, and a negative amplitude for nuclear export, $G_{c,\mathrm{sub,exp}}(\tau )$. **(H)** Fluorescence image shown in A (right) with numbers designating positions along the nuclear envelope where two-foci cross-correlation analysis was performed, and arrows showing the directions of mStayGold-GR nucleocytoplasmic translocation: nuclear import (red) and nuclear export (blue). Scale bar: 10 μm.

In our previous work, eGFP-GR nucleocytoplasmic shuttling was characterized in live cells at different positions along the nuclear envelope 15 min after treatment with 500 nM Dex, *i.e.*, at the half time of ligand-induced eGFP-GR nuclear import [22]. Here, we characterized mStayGold-GR nucleocytoplasmic translocation using mpFCS (Fig. 5). Under the same conditions as before, 15 min after initiating the treatment with 500 nM Dex, mStayGold-GR has largely translocated into the cell nucleus (Fig. 5A, left), whereas a comparatively small amount of mStayGold-GR was still present in the cytoplasm, as can be seen in the highly contrasted image (Fig. 5A, right). Using mpFCS, we could simultaneously record fluorescence intensity fluctuations at different locations in the cytoplasm and the cell nucleus, obtaining ACCs at all locations (Fig. 5B). As expected, the average ACC recorded in the cell nucleus was characterized by significantly longer decay time than the one recorded in the cytoplasm (Fig. 5C), consistent with GR binding to the genomic DNA. Spatial mapping of diffusion times (Fig. 5D) clearly showed longer diffusion times, *i.e.*, slower mobility. In the cytoplasm, there was no statistically significant difference between the diffusion coefficients of the two components measured by spFCS in untreated cells and mpFCS in Dex-treated cells (Fig. 5E, left), which is consistent with our previous results showing that the effective volume in the spFCS and mpFCS systems are similar [21]. However, the relative fraction of the second component was significantly higher in treated *vs* untreated cells (Fig. 5E, right), consistent with GR binding to cytoplasmic structures (*e.g.*, microtubles) and/or protein complexes (*e.g.*, transporting machinery). Both spFCS and mpFCS showed that mStayGold-GR diffusion in the cell nucleus is complex, identifying at least three characteristic decay times, *i.e.*, three components. Fixing the diffusion time of the first component as described in the Methods section, diffusion times of the second/third component could be determined, yielding, as in the cytoplasm, similar values for spFCS and mpFCS measurements (Fig. 5F, left). While statistically significant difference was not observed for the fraction of the first component between the spFCS and mpFCS measurements, the relative amplitude of the second/third components were significantly different (Fig. 5F, right). To understand whether this difference is arising due to differences between spFCS, for which the data dispersion is reflecting differences between different cells, and mpFCS, which reflects intracellular differences, we have analyzed mpFCS data acquired on different cells (Fig. S15). This analysis suggests that the stepwise decay observed at long lag times, τ≈1s (red; Fig. 5C), is due to low data sampling at such long lag times in a time series that is acquired for 40 s, may contribute to these differences. In line with this, the difference in relative molar fractions arises due to the offset, the value of which needs to be estimated using the data fitting. Of note, a large spread of measured values is observed, suggesting that heterogeneity in the availability of DNA-binding sites within a single cell or among cells is important.

Two-foci cross-correlation (tfCCC) analysis ($G_{c}(\tau )$) used to characterize the direction of GR translocation and the passing time between the cytoplasm and the nucleus along the nuclear envelope (Fig. 5G), yielded the so-called subtracted CCCs, $G_{c,\mathrm{sub}}(\tau )=G_{c,\mathrm{imp}}(\tau )\;-\;G_{c,\exp}(\tau )$, showing the direction of nuclear import/export as positive/negative peaks, respectively. Importantly, tfCCC with zero amplitude, *i.e.*, no tfCCC, was observed in cells expressing the fluorescent protein mStayGold. Nuclear import of mStayGold-GR was observed at sites: 2, 3, 4, 7, 8, 9, 10, 11, and 13 (Fig. 5H, red), whereas nuclear export of mStayGold-GR was observed at sites: 1, 5, 6, 12, 14, and 15 (Fig. 5H, blue). Integration of the subtracted CCCs clearly showed a curve with a positive peak (Fig. S16, black), indicating that mStayGold-GR is still being imported into the cell nucleus. Peaks in the subtracted CCCs occurred at lag times of (10–20) s, consistent with our previous data for eGFP-GR showing a peak width of (1–20) s. In contrast, mStayGold-GR improved fitting precision (Fig. S17). Also, peaks with extended width or a complex peak structure with multiple components are indicative of transport in both directions or through several nuclear pore complexes concomitantly observed in the same OVE. For example, a random walk characterized by alternating forward and backward motions can produce wider peaks, with peak-width depending on the frequency and extent of the directional changes.

## 4. Discussion

Photobleaching is a persistent challenge in fluorescence microscopy/correlation spectroscopy experiments. eGFP is widely used in live-cell fluorescence microscopy due to its brightness, photostability, monomeric size and absence of post-translational modifications. However, despite its relative photostability compared to other fluorescent proteins, eGFP still suffers from photobleaching during live-cell microscopy experiments.

To address this issue, brighter variants such as mNeonGreen have been developed [60,61], but photobleaching remained a limitation. Recently, a photostable dimeric GFP known as StayGold was introduced [42]. StayGold offers superior brightness and exceptional resistance to photobleaching. However, as a dimeric fluorescent tag, it can interfere with the functionality of the target protein. Hence, several monomeric variants of StayGold have been developed, including mStayGold, StayGold/E138D, and mBaojin [43–45]. These monomeric variants retain the brightness and photostability of the original dimeric StayGold, while minimizing potential functional disruptions to the protein of interest.

In this study, we have characterized the suitability of mStayGold and StayGold/E138D for conventional spFCS and mpFCS in live cells, which require prolonged recording of fluorescence intensity fluctuations leading likely to photobleaching even under low-intensity illumination. Photobleaching introduces an additional component in the ACC, with a long decay time that is typically in the (1–10) s range, causing underestimation of the number of molecules (due to an artificially large amplitude of the ACC) and “masks” the diffusion of slow-moving molecules.

To mitigate photobleaching effects, pre-bleaching before FCS experiments is typically used and/or photobleaching effects are accounted for in the analysis of fluorescence intensity fluctuations by incorporating photobleaching effects into fitting models. This may be done either by adding an offset for correlation at infinite time or by including photobleaching kinetics by adding an exponential decay curve in the data fitting function, as photobleaching typically follows an exponential decay [39,40]. Frequency-domain analysis, leveraging the low-frequency nature of photobleaching can also be used [41]. While these techniques are effective, they require additional processing of fluorescence intensity fluctuation data *post* data acquisition. They carry also significant risks – the possibility to underestimate the number of molecules, *i.e.*, the concentration of molecules of interest, and to lose information about slower diffusion components.

Our results from both spFCS and mpFCS demonstrate that mStayGold and StayGold/E138D are twice as bright as eGFP and exhibit minimal photobleaching during spFCS and mpFCS measurements, enabling measurements with high SNR.

Using mStayGold, we analyzed in live cells the dissociation constant for the homodimerization of wild-type GR genetically fused with eGFP or mStayGold. Molecular brightness measurements yielded values consistent with previous results obtained using fluorescence cross-correlation spectroscopy (FCCS) [32]. Notably, the relative standard deviation was smaller with mStayGold, indicating that its high signal-to-noise ratio enhances the precision of molecular brightness measurements, resulting in reduced variability in the dissociation constant. This improved precision may lower the number of data points required for accurate dissociation constant analysis. Furthermore, better precision in molecular brightness can compensate for variability introduced by heterogeneity in genomic DNA organization in different parts of the same cell and among different cells.

We further demonstrated molecular transport analysis using cross-correlation between two foci: one in the cytoplasm and another in the nucleus. Overall, mStayGold-GR was imported into the nucleus during time-lapse observations under Dex treatment. Interestingly, the translocation of mStayGold-GR at individual positions was location-specific, aligning well with our previous findings using eGFP-GR [22]. Notably, photobleaching correction was unnecessary with mStayGold-GR, streamlining data analysis and saving time and effort.

## 5. Conclusion

In summary, this work shows that mStayGold and StayGold/E138D are under the conditions relevant for this experiment twice as bright as eGFP and more photostable. This makes it possible to acquire photobleaching-free spFCS and mpFCS data even for longer measurement times, thereby abolishing the need for FCS data pre-processing to account for photobleaching. These advances are crucial for addressing the technical limitations that hinder our understanding of how complex biological functions such as gene expression emerge in live cells through entangled reaction-diffusion networks. The bold vision to create “virtual cells” [62] is constrained by the availability of high-resolution, quantitative tools to capture without disruption the fast cellular dynamics of molecules. mpFCS is a novel dynamic imaging method that eliminates the need for scanning, while retaining the capacity of optical sectioning of confocal laser scanning microscopy. By delivering spatially resolved quantitative maps of molecular concentration, diffusion, local environment, and direction of molecular motion with single-molecule sensitivity in live cells, mpFCS provides critical information needed for understanding the dynamic integration of molecular interactions in live cells. This will enable researchers to progress in their efforts towards emulating *in silico* self-regulation and the emergence of biological functions that are characteristic of the smallest unit of animate matter, the cell.

Moreover, these new tools will enable a more detailed investigation of the cellular dynamics of glucocorticoid receptors and their mechanisms of action, focusing on yet unresolved challenges associated with glucocorticoid pharmacotherapy, such as glucocorticoid insensitivity, whether genetically inherited or acquired through environmental or physiological changes, and treatment side effects, like metabolic disturbances, immune suppression, or tissue-specific resistance.

## Supplementary Material

1

## Data Availability

Data supporting this study is included in the article and/or supporting materials. Raw data are available on request.

## Data availability

Data will be made available on request.

## Appendix A. Supplementary data

Supplementary data to this article can be found online at https://doi.org/10.1016/j.bbagen.2025.130809.
