# Supplementary material for: Celebrating 50 years of fluorescence correlation spectroscopy (FCS): Advancing live-cell massively parallel FCS studies with photostable GFPs, mStayGold and StayGold/E138D
Source: Biochim Biophys Acta Gen Subj. Author manuscript; Available in PMC 2026 Jun 18. (PMC13276660; doi:10.1016/j.bbagen.2025.130809)
Supplement: 1 [file NIHMS2174812-supplement-1.pdf]

Supplementary Information:

Celebrating 50 Years of fluorescence correlation spectroscopy (FCS): Advancing live-cell massively parallel FCS studies with photostable GFPs, mStayGold and StayGold/E138D

Sho Oasa*, Borislav Stoyanov, Yuta Hamada, Stanko N. Nikolić, Aleksandar J. Krmpot, Akira Kitamura, Lars Terenius and Vladana Vukojević*

*Corresponding authors

Supplementary methods

Fluorescence intensity correction

Fluorescence intensity traces, with temporal resolution of 40 μs/frame, were binned into 400 μs. A 4th-order polynomial function was used to fit the fluorescence intensity traces, and they were corrected [1-3]:

$$I_{cor}(t) = \frac{I(t)}{\sqrt{\frac{f(t)}{f(0)}}} + f(0) \cdot \left(1 - \sqrt{\frac{f(t)}{f(0)}}\right) \qquad \text{(S1)}$$

Here, $I(t)$ and $I_{cor}(t)$ are fluorescence intensity of raw and photobleaching-corrected data at time t, respectively; and $f(0)$ and $f(t)$ denote values of the 4th-order polynomial function at time 0 and t, respectively.

Autocorrelation curves from uncorrected and corrected fluorescence intensity fluctuations (Fig. S6C) were calculated using the Origin Data Analysis and Graphing software (OriginLab).

Multi-component Gaussian model fitting to tfCCCs

The subtracted tfCCC was analyzed via the multi-component gaussian model in the Origin Data Analysis and Graphing software:

$$G_{\mathrm{c,sub}}(\tau) = G(\infty) + \sum_{q=1}^{n} \frac{S_q}{w_q \cdot \sqrt{\frac{\pi}{2}}} \cdot e^{\left(\frac{-2\cdot(\tau-\tau_{trans,q})^2}{w_q^2}\right)} \qquad (S2)$$

where, $G(\infty)$ denotes the offset of cross-correlation at unlimited time. S, w and τ_{trans} are an area, width and peak time of the Gaussian model. The n is the number of components which was set as 1 or 2.

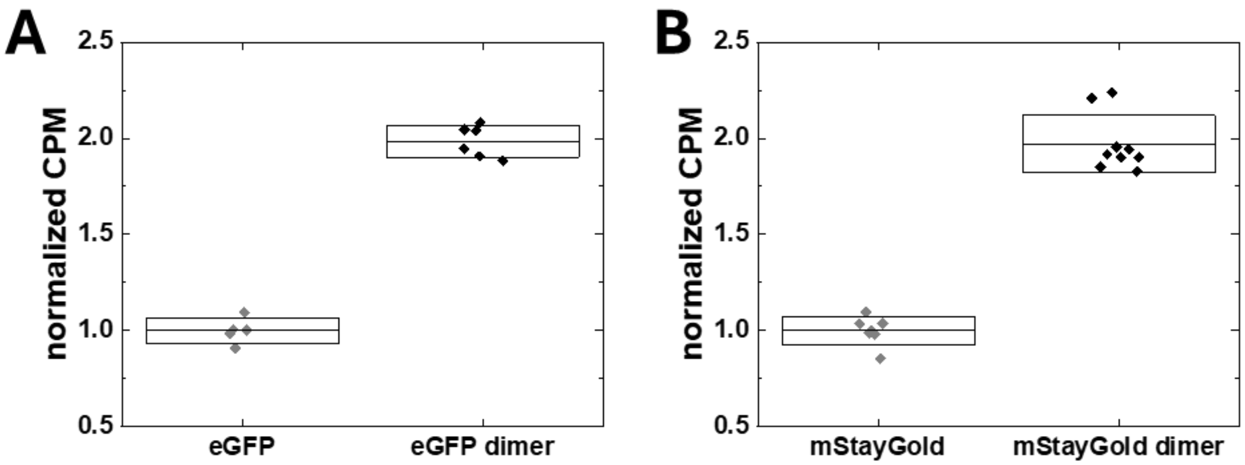

Figure S1. eGFP and mStayGold tandem dimers are twice brighter, as reflected by CPM, than the corresponding monomeric fluorescent protein variants. (A, B) CPM of eGFP and eGFP dimer (A) and mStayGold and mStayGold dimer (B), normalized by CPM of monomeric eGFP (A) and mStayGold (B). The normalized CPM values, given as Average ± Standard Deviation, are for monomeric eGFP: 1.00 ± 0.07, and for tandem dimer of eGFP: 1.98 ± 0.08. The corresponding values for mStayGold are for monomeric mStayGold: 1.00 ± 0.07, and tandem dimer of mStayGold: 1.97 ± 0.15. The data suggests that the contribution of fluorescent proteins' dark states in the homodimer has a negligible effect on their brightness.

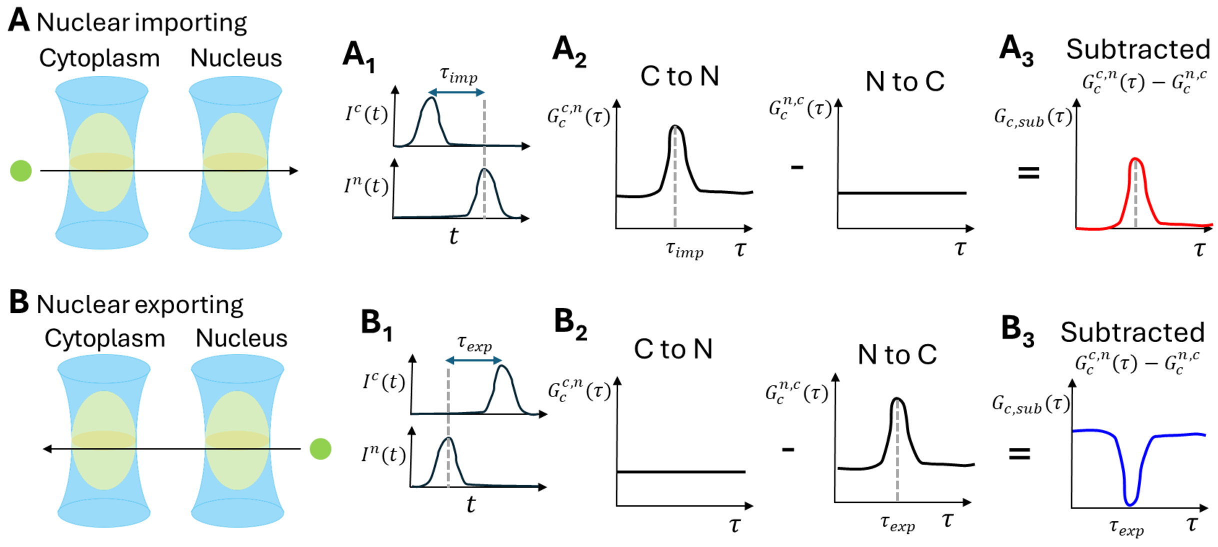

Figure S2. Schematic diagram showing the procedure of two-foci cross-correlation curve (tfCCC) calculation for molecular transport characterization. (A, B) Transport of one molecule from an effective volume element in the cytoplasm to the one in the cell nucleus, reflecting nuclear import (A) and transport in the opposite direction, reflecting nuclear export (B). **(A1, B1)** Fluorescence intensity in the cytoplasm, $I^c(t)$, and the nucleus, $I^n(t)$, shows changes in fluorescence intensity generated by the molecule passing through. The detected fluctuations are shifted in time with respect to one another, τ_{imp} and τ_{exp} for nuclear import and export, respectively, reflecting the time it takes for the molecule to pass through both effective volume elements. **(A2, B2)** In the tfCCC calculated in the direction of molecular motion, from the cytoplasm to the cell nucleus, $G_c^{c,n}(\tau)$, a peak is observed at τ_{imp} (A2). Likewise, it is observed at τ_{exp} for the tfCCC, $G_c^{n,c}(\tau)$. In contrast, no peak is observed when the tfCCCs are calculated in the direction that is opposite from the direction of molecular motion. **(A3, B3)** The subtracted tfCCC, $G_{c,sub}(\tau) = G_c^{c,n}(\tau) - G_c^{n,c}(\tau)$, readily visualizes the translocation direction as positive for nuclear import or negative for nuclear export.

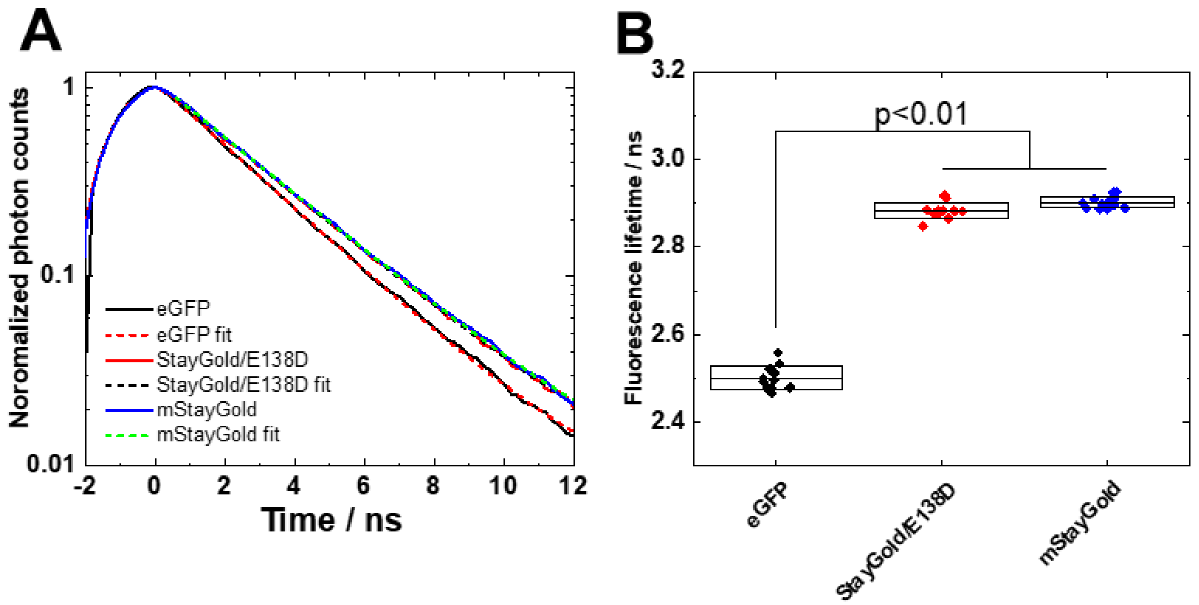

Figure S3. Fluorescence lifetime of eGFP, StayGold/E138D and mStayGold in live HEK cells measured using massively parallel fluorescence correlation spectroscopy integrated with fluorescence lifetime imaging microscopy (mpFCS/FLIM). (A) FLIM curves normalized to the same amplitude, $I_n(t) = 1$ at $t = 0$, using maximum photon counts as a reference. Best-fit curves using a single-component exponential decay model are shown as dashed lines. Black full line with red dashed line: eGFP; Red full line with black-solid line: StayGold/E138D; Blue full line with green dashed line: mStayGold. **(B)** Mean fluorescence lifetime and standard deviation for each GFP variant, calculated from 10 individual cells. The middle line and boxes represent the sample mean and standard deviation, respectively. Black: eGFP; Red: StayGold/E138D; Blue: mStayGold. Statistical analysis was conducted using one-way ANOVA with a *post hoc* Tukey test.

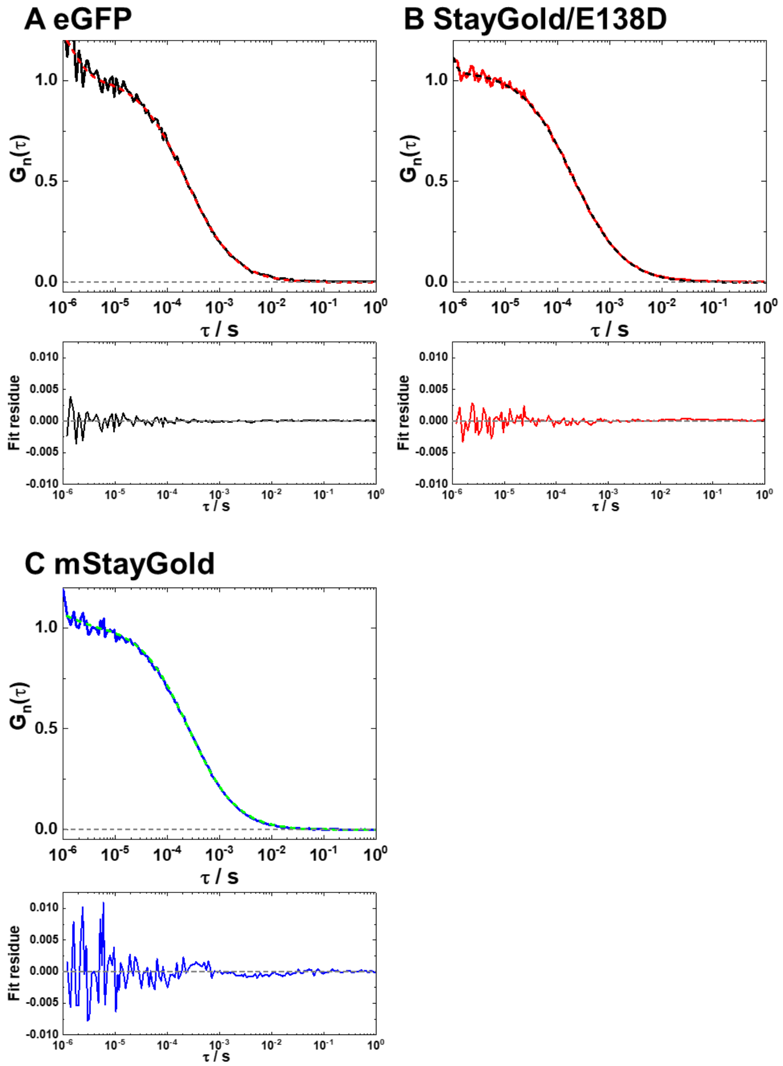

Figure S4. Autocorrelation curves recorded in HEK cells expressing analyzed GFP variants. Temporal autocorrelation curves (tACCs) normalized to the same amplitude, $G_n(\tau) = 1$ at $\tau = 10$ μs, (top) and corresponding fit residuals (bottom) for: **(A)** eGFP, **(B)** StayGold/E138D, and **(C)** mStaygold. Dashed lines represent best-fit curves obtained using the single-component anomalous diffusion model. Fit residuals represent the difference between the autocorrelation curve and its fitted curve at each time delay, *i.e.*, lag time τ.

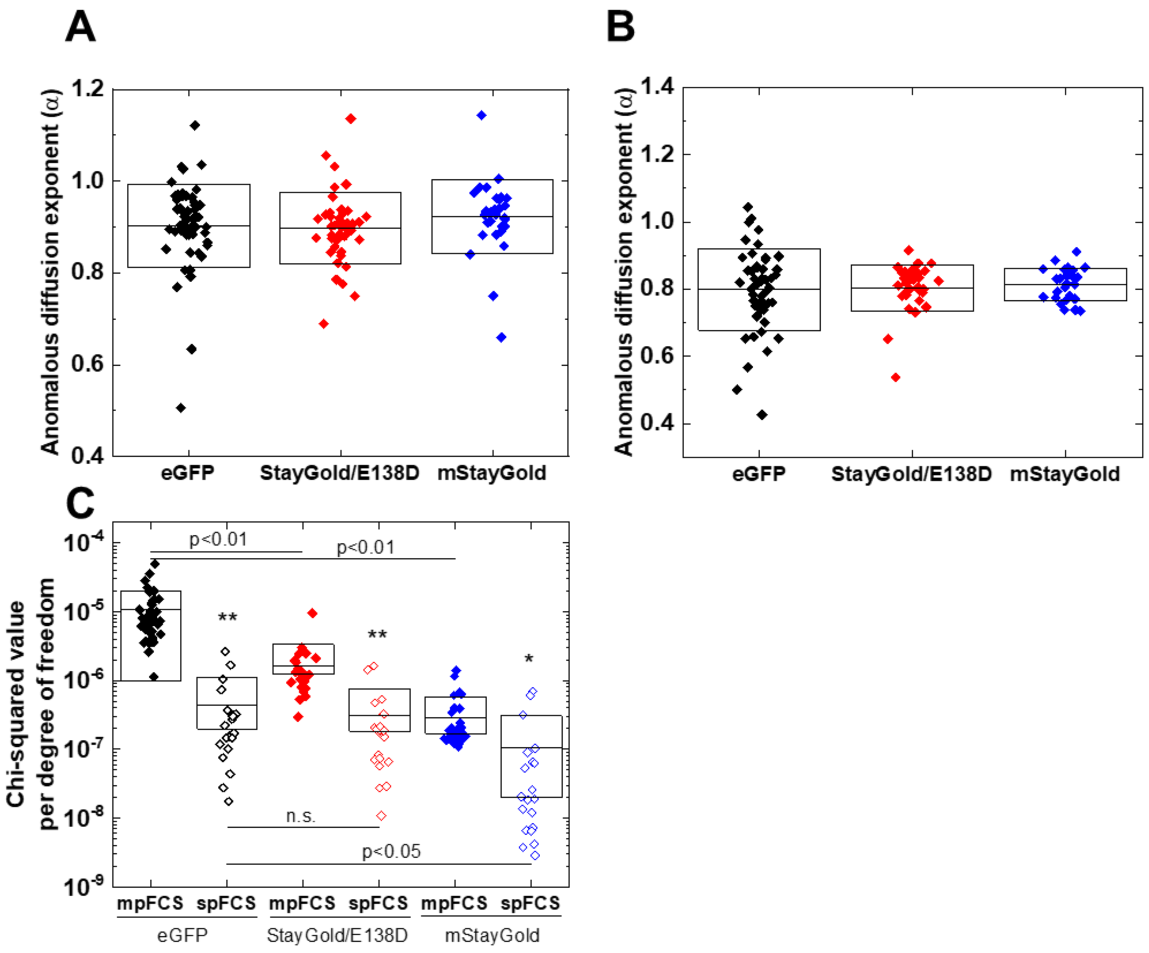

Figure S5. No significant changes in anomalous diffusion exponent were observed between eGFP, StayGold/E138D and mStayGold. (A, B) Average ± Standard Deviation of anomalous diffusion exponent characterized by spFCS (A) and mpFCS (B). The one-way ANOVA with a *post hoc* Tukey test indicates no significant differences between GFP variants ($p > 0.05$). **(C)** Average ± Standard Deviation of chi-square values *per* degree of freedom. Filled plots: mpFCS, Open plots: spFCS. Black: eGFP, Red: StayGold/E138D, Blue: mStayGold. The two-tailed Student's t-test was performed between eGFP and GFP variants or between mpFCS and spFCS (*$p < 0.05$, **$p < 0.01$).

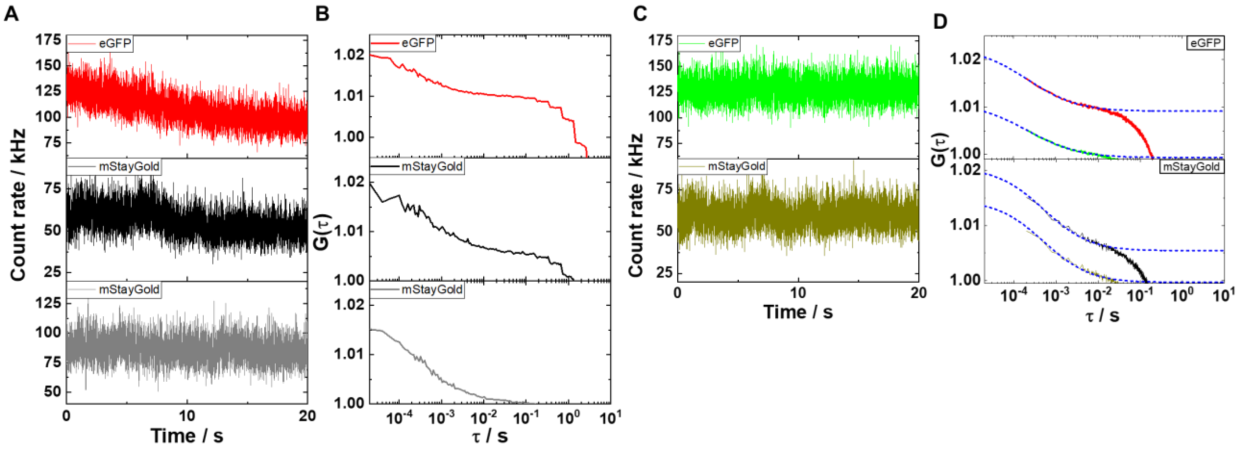

Figure S6. Correcting the decay in fluorescence intensity due to photobleaching and baseline instability yields improved autocorrelation curves devoid of contributions from these unwanted processes. (A, B) Original fluorescence intensity fluctuations (A) and corresponding autocorrelation curves (B) for eGFP with photobleaching (red), mStayGold with an unexpected drop in average fluorescence intensity (black) and an original time series for mStayGold without any such contributions (Grey). Photobleaching of eGFP and the unexpected drop in average fluorescence intensity for mStayGold, generated an additional component in the corresponding autocorrelation curve, with a characteristic decay time value of around 1 s. Temporal autocorrelation analysis of the mStayGold time series without any such changes in fluorescence intensity, did not show the second decay component. **(C, D)** Average fluorescence intensity decay correction using the 4th-order polynomial function (Eq. S1) yielded corrected fluorescence intensity traces for eGFP (light green) and mStayGold (green) (C). Temporal autocorrelation analysis of the corrected time series resulted in each case the disappearance of the second component in the tACC characterized by the decay time of around 1 s (D). Red and light green tACCs derived for eGFP from time series without and with fluorescence intensity correction. Black and green tACCs derived for mStayGold from time series without and with fluorescence intensity correction. Blue: fitting curve obtained in (B) with different offset at infinite lag time, $G(\infty)$ in the fitting equation. This suggests that photobleaching and unexpected changes in the average fluorescence intensity generate an additional component in the temporal autocorrelation curve with a characteristic decay time of around 1 s, but not affect the autocorrelation curve that is a characteristic of diffusion, with a characteristic decay time of around 300-500 μs.

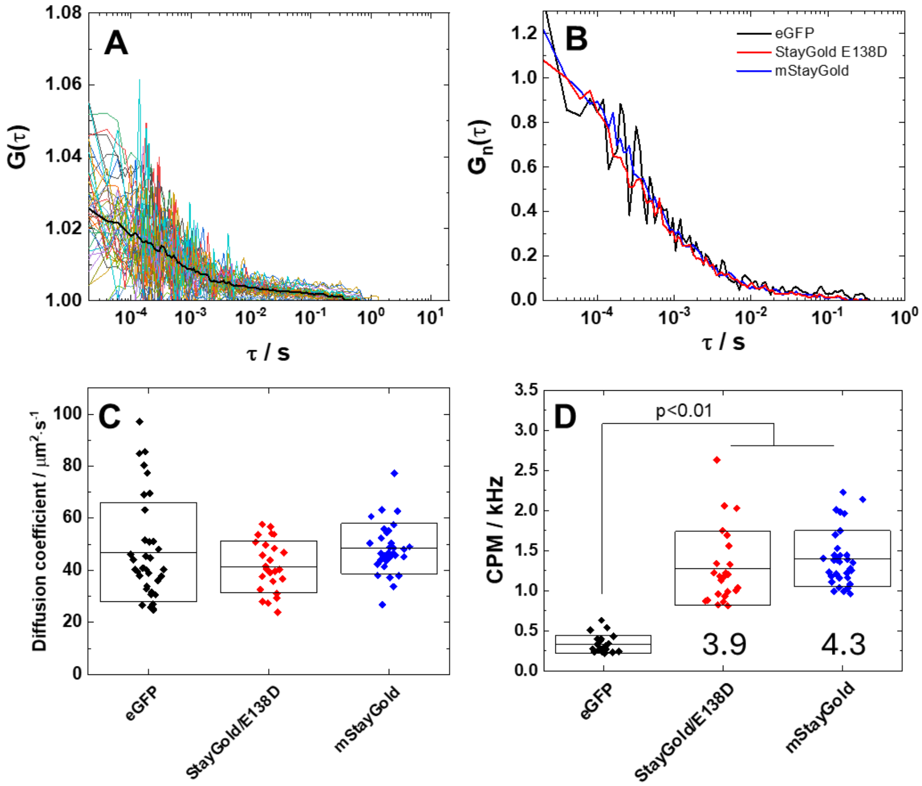

Figure S7. StayGold/E138D and mStayGold exhibit a high signal-to-noise ratio and increased the molecular brightness. (A) Temporal autocorrelation curves (tACCs) recorded in live HEK cells expressing eGFP. When acquired after pre-bleaching, distortion of ACCs due to eGFP photobleaching is no longer observed. The thick black line represents the average ACC. The thin full line reflects single-pixel autocorrelation curves acquired using mpFCS. **(B)** Single-pixel ACCs normalized to the same amplitude, $G_n(\tau) = 1$ at $\tau = 20$ μs, recorded in live eGFP-expressing cells after pre-bleaching (black) and in StayGold/E138D- (red) and mStayGold-expressing cells (blue) acquired without any pre-bleaching. **(C)** Diffusion coefficient of eGFP, StayGold/E138D and mStayGold. **(D)** Apparent brightness of eGFP, StayGold/E138D and mStayGold, reflected through counts *per* second *per* molecule (CPM). In the boxplots (C and D), the middle line and boxes indicate the sample mean value and the standard deviation, respectively. Apparent molecular brightness of mStayGold and StayGold/E138D relative to that of eGFP (Eq. 10), *i.e.*, relative molecular brightness gives $R = 3.9$ for StayGold/E138D and $R = 4.3$ for mStayGold. Statistical analysis was conducted using one-way ANOVA with a *post hoc* Tukey test. Lack of statistically significant difference is denoted as n.s., $p > 0.05$.

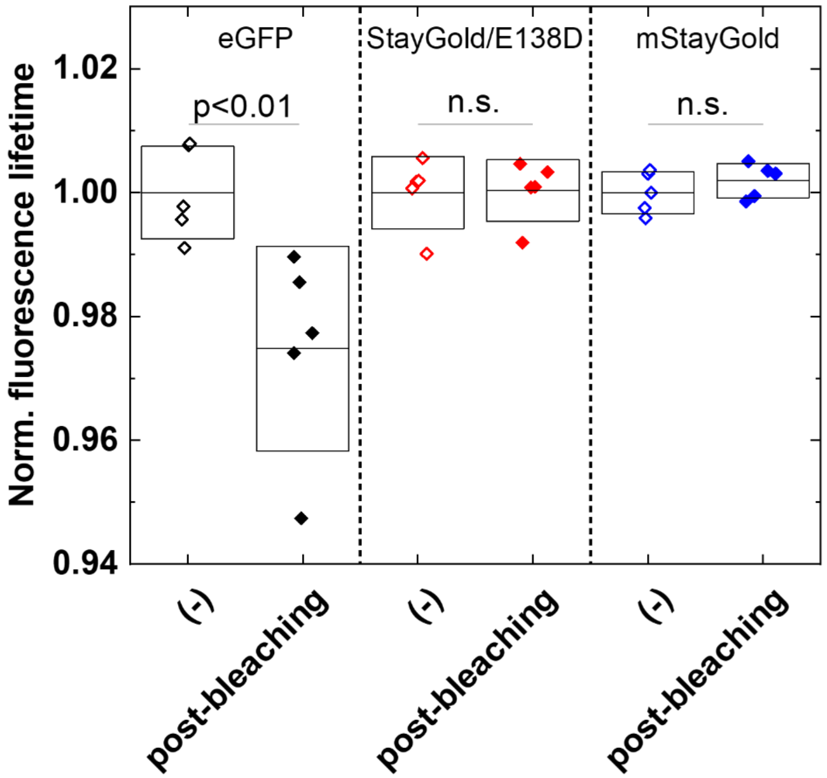

Figure S8. Dynamic quenching may contribute to underestimation of CPM of eGFP in mpFCS measurements. Average ± Standard Deviation of fluorescence lifetime (FL) normalized by that before the pre-bleaching. FL was significantly shortened by the 100-second pre-bleaching in eGFP-expressed cells (Black), but no such change was observed in StayGold/E138D- and mStayGold-expressed cells (Red and Blue). Open plots: before bleaching, Filled plots: post-bleaching. The two-tailed student's t-test was performed. No significant difference ($p > 0.05$) represents as n.s.

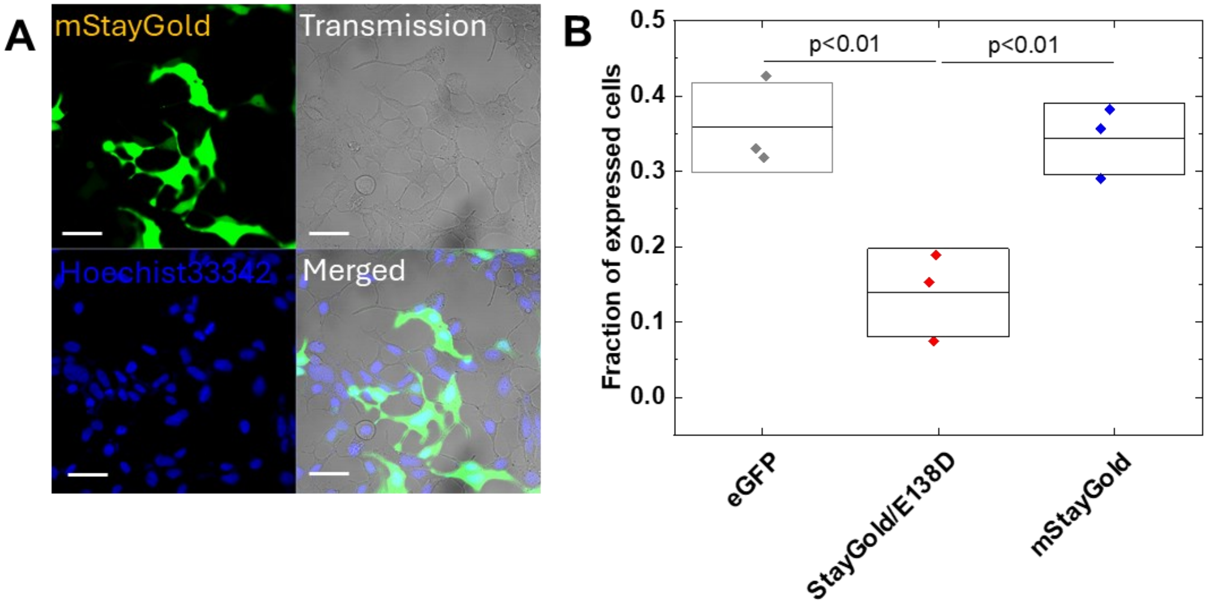

Figure S9. mStayGold shows similar transfection efficiency as eGFP in HEK cells. (A) Typical confocal laser scanning fluorescence microscopy image of HEK cells transiently expressing mStayGold (green), co-staining with Hoechst33342 (blue). Scale bar: 20 µm. **(B)** Fraction of mStayGold-expressing cells (F_{exp}) was calculated as the ratio of the number of green fluorescently positive cells (N_p) and total number of cells assessed *via* the number of nuclei (N_n) ($F_{exp} = N_p / N_n$). Statistical analysis was conducted using one-way ANOVA with a *post hoc* Tukey test. Lack of statistically significant difference is denoted as n.s., $p > 0.05$.

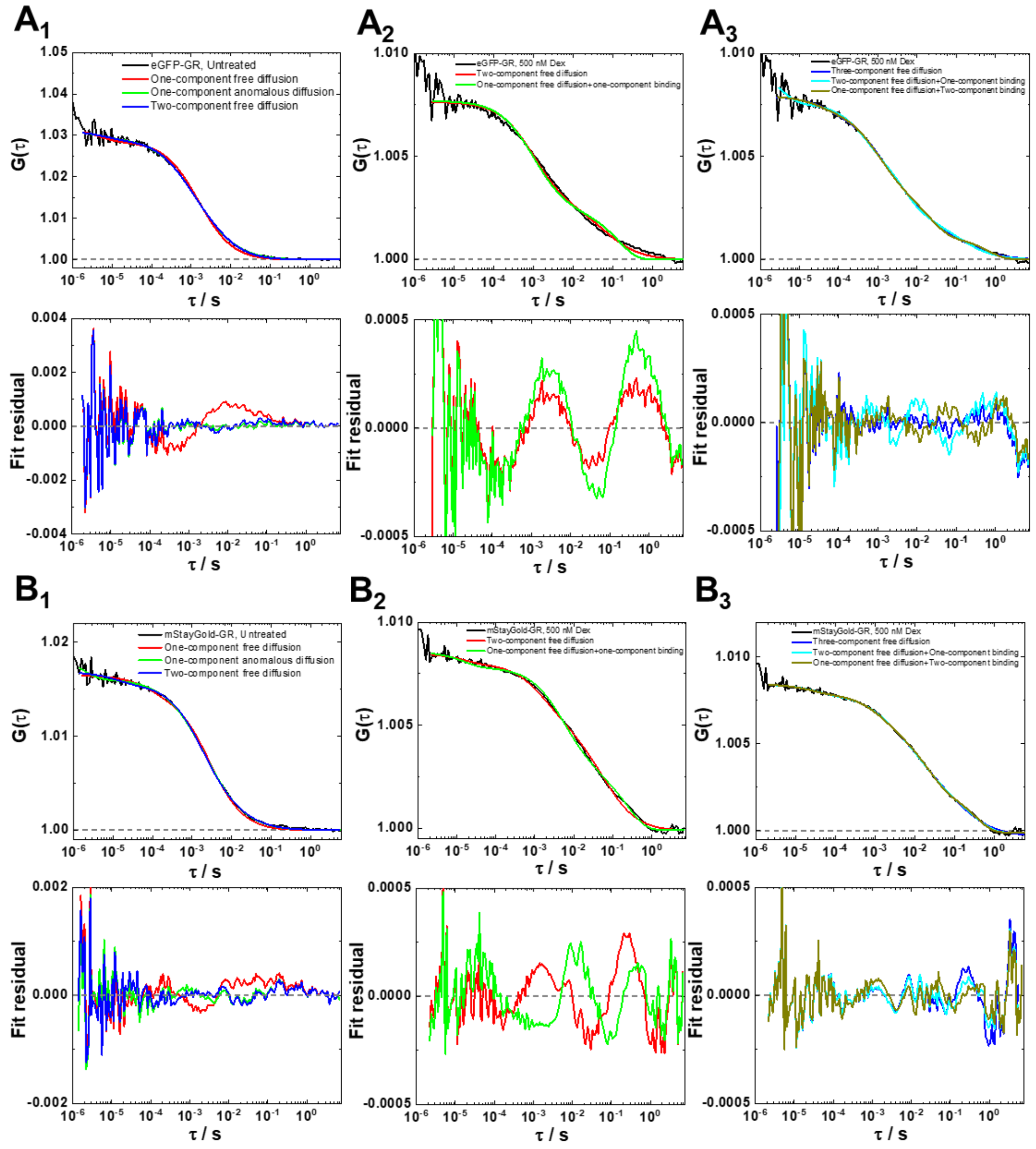

Figure S10. Analysis of temporal autocorrelation curves (tACCs) and fitting model selection for characterizing eGFP-GR and mStayGold-GR cellular dynamics and interactions in live HEK cells. (A_1, B_1) ACCs fitting (top) and fit residuals (bottom) for eGFP-GR (A_1) and mStayGold-GR (B_1) recorded in the cytoplasm of untreated HEK cells. Red: Single-component free diffusion model; Green: Single-component anomalous diffusion model; Blue: Two-component free diffusion model. **(A_2, B_2)** ACCs fitting using two-component models (top) and fit residuals

(bottom) for eGFP-GR (A_2) and mStayGold-GR (B_2) recorded in the nucleus of cells treated for 30 min with 500 nM Dex. Red: Two-component free diffusion model; Green: One-component free diffusion + One-component binding model. **(A_3, B_3)** ACCs fitting using three-component models (top) and fit residuals (bottom) for eGFP-GR (A_3) and mStayGold-GR (B_3) recorded in the nucleus of cells treated for 30 min with 500 nM Dex. Blue: three-component free diffusion model; Light blue: two-component free diffusion + one-component binding model; Dark green: one-component free diffusion + two-component binding model. Given the lack of differences between the one-component anomalous diffusion model and the two-component free diffusion model for fitting ACCs recorded in the cytoplasm of untreated cells, the two-component free diffusion model was adopted. Likewise, given the lack of difference between the three-component models in the cell nucleus, the three-component free diffusion model was chosen for fluorescence intensity fluctuations analysis in the nucleus of cells treated for 30 min with 500 nM Dex.

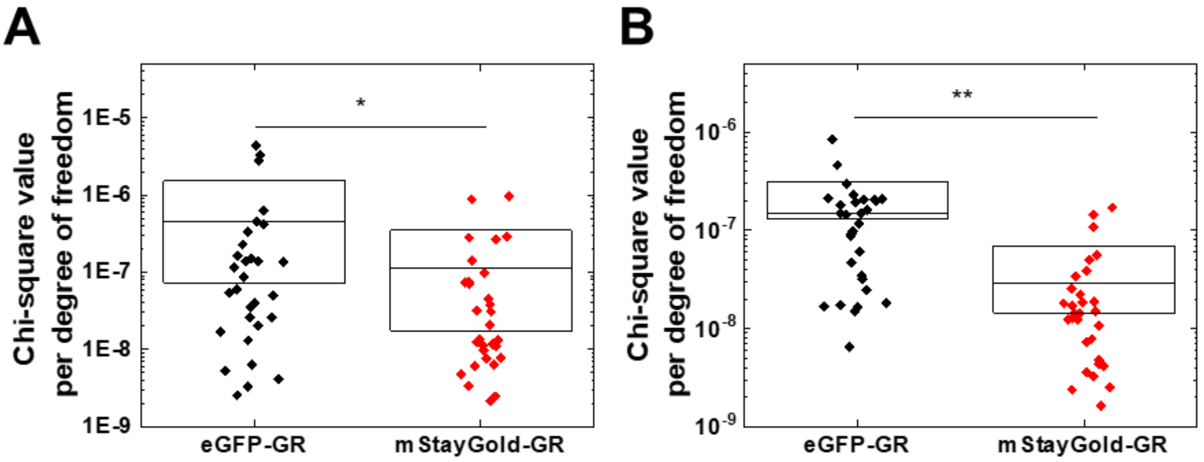

Figure S11. Analysis of autocorrelation curves fitting precision. Chi-square values *per* degree of freedom were calculated for autocorrelation curves (ACCs) fitting using: **(A)** the two-component free diffusion model for fitting ACCs recorded in the cytoplasm of untreated HEK cells, and **(B)** the three-component free diffusion model for fitting ACCs recorded in the nucleus of cells treated for 30 min with 500 nM Dex. Statistical analysis was performed using a two-tailed Student's t-test. Statistical significance: *$p < 0.05$, **$p < 0.01$.

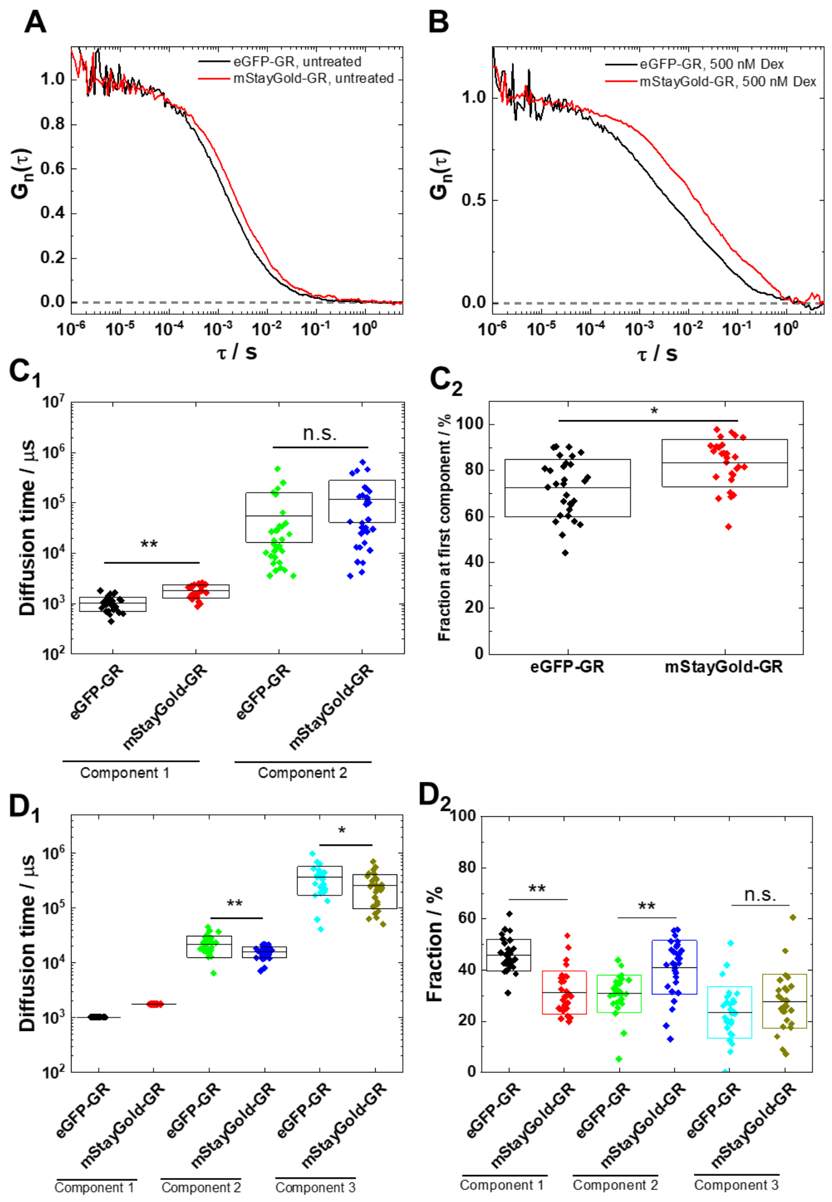

Figure S12. Comparison of eGFP-GR and mStayGold-GR diffusion properties. (A, B) Autocorrelation curves (ACCs) normalized to the same amplitude, $G_n(\tau) = 1$ at $\tau = 10$ µs. Black: eGFP-GR, Red: mStayGold-GR. **(C_1, C_2)** Diffusion time of the first and the second component (C_1) and the relative molar fraction (F_1) of the first component (C_2) for eGFP-GR and mStayGold-GR in the cytoplasm of untreated cells. **(D_1, D_2)** Diffusion time (D_1) and the relative molar fraction (D_2) when using the three-component model for eGFP-GR and mStayGold-GR in the nucleus of cells treated for 30 min with 500 nM Dex. Statistical analysis was performed using a two-tailed Student's t-test. Statistical significance: n.s. when $p > 0.05$. *$p < 0.05$, **$p < 0.01$.

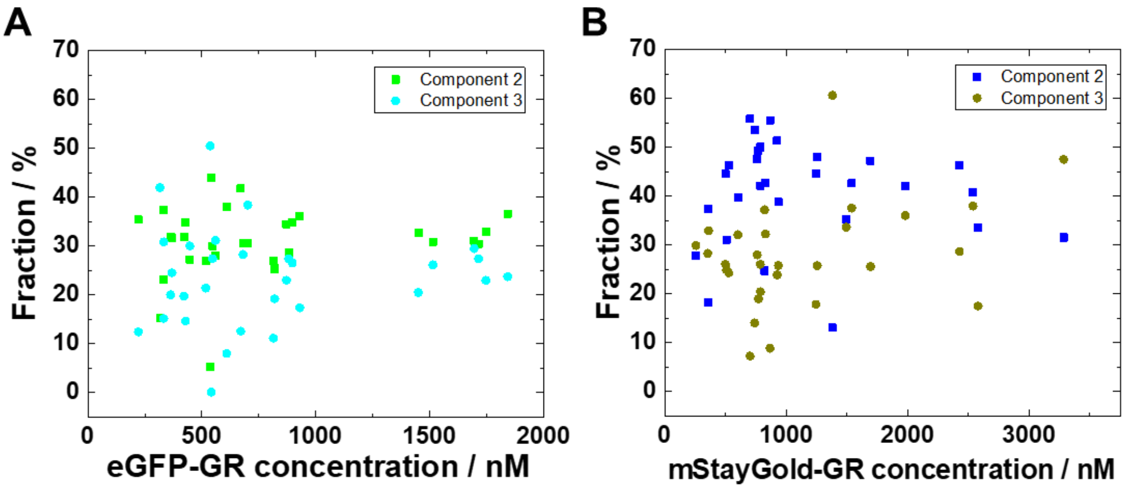

Figure 13. Scatter plots showing eGFP-GR and mStayGold-GR DNA-binding-fractions as a function of their respective concentrations. (A) Fractional percentage of eGFP-GR-DNA-binding-related components (second and third components). **(B)** Fractional percentage of mStayGold-GR-DNA-binding-related components (second and third components).

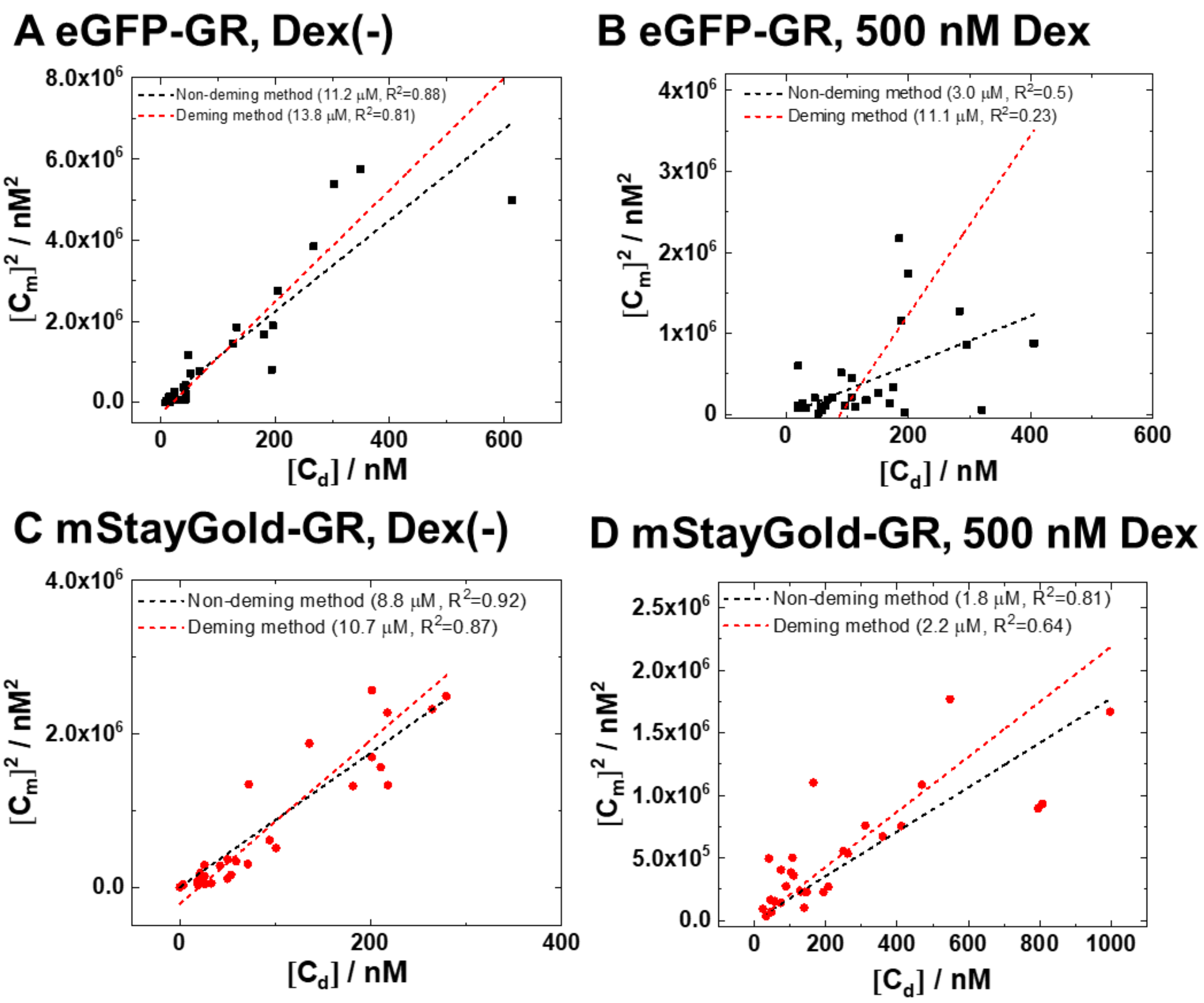

Figure S14. Comparison of linear regression analysis between non-Deming (NDem) and Deming (Dem) methods. (A, B) Linear regression analysis using both methods determined the dissociation constant of eGFP-GR homodimerization to be $K_{d,homo}^{NDem}$ = 11.2 μM and $K_{d,homo}^{Dem}$ = 13.8 μM in untreated cells (A), and $K_{d,homo,Dex}^{NDem}$ = 3.0 μM and $K_{d,homo,Dex}^{Dem}$ = 11.1 μM in cells treated with 500 nM Dex (B). **(C, D)** Linear regression analysis using both methods determined the dissociation constant of mStayGold-GR homodimerization to be $K_{d,homo}^{NDem}$ 8.8 μM and $K_{d,homo}^{Dem}$= 10.7 μM in untreated cells (C) and $K_{d,homo,Dex}^{NDem}$ =1.8 μM and $K_{d,homo,Dex}^{Dem}$ = 2.2 μM in 500 nM Dex-treated cells (D). The non-Deming *vs* Deming methods did not make remarkable differences in cases shown in A, C, and D, whereas the Deming method did not work in the case of eGFP-GR homodimerization under the treatment with 500 nM Dex due to the large scattering data. Even in cells treated with 500 nM Dex, mStayGold-GR reduced data scattering, enabling more accurate and precise dissociation constant determination.

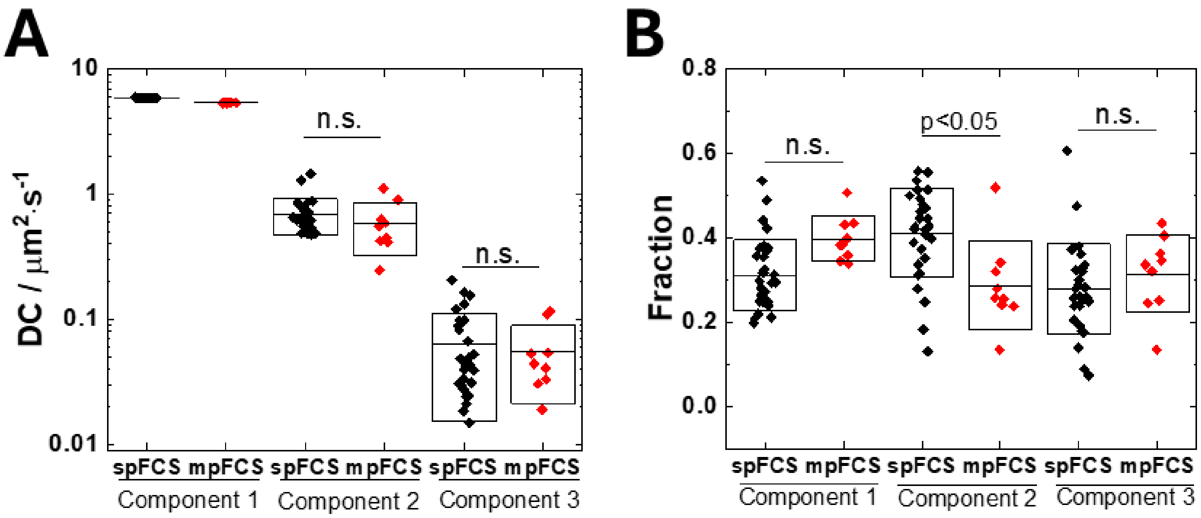

Figure S15. Comparison of diffusion coefficients and the relative molar fractions of different components in different cells measured by spFCS and mpFCS. (A, B) Diffusion coefficients (A) and relative molar fractions (B) measured in different cells by spFCS (black) and mpFCS (red). Statistical analysis was performed by the two-tailed student's t-test. No statistically significant difference n.s. indicates $p > 0.05$. The data confirmed that statistically significant differences was not observed for diffusion coefficients of the second and third component, nor in the relative molar fraction of the third component. However, the relative molar fraction of the second component remained significantly different, while the relative molar fraction of the first component showed a difference that was not statistically significantly different. This data may suggest that the cell population heterogeneity (*e.g.*, co-factor expression) is not the primary cause of the differences between spFCS and mpFCS.

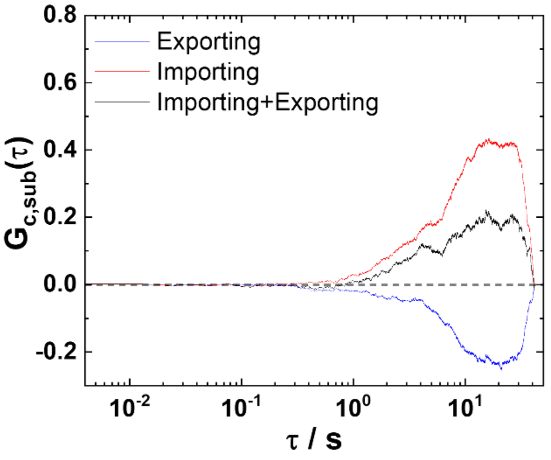

Figure S16. The integral subtracted cross-correlation curve for mStayGold-GR import/export in HEK cell obtained by two-foci cross-correlation analysis. Summation of two-foci cross-correlation curves shown in Fig. 5. Blue: export, Red: import, Black: import + export. Black spCCC shows a positive peak, indicating that mStayGold-GR is, in total, imported into the nucleus.

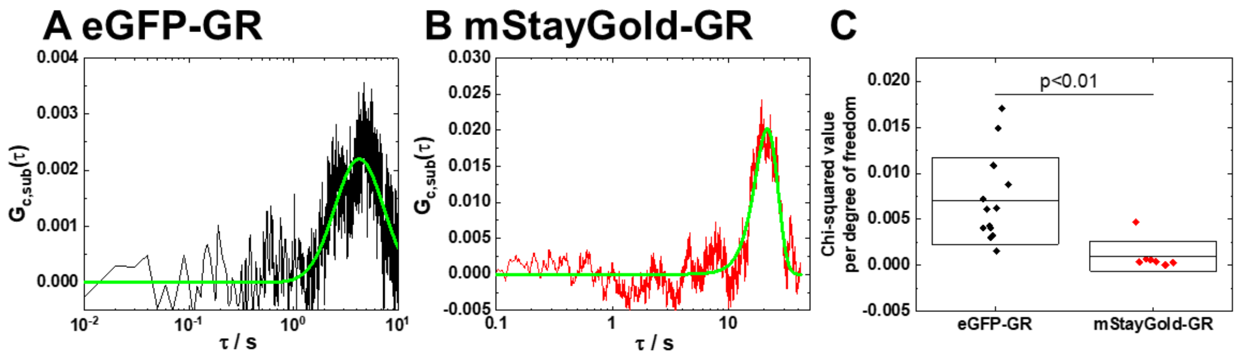

Figure S17. Comparison of tfCCCs data fitting precision between eGFP-GR and mStayGold-GR. (A, B) Typical tfCCC in eGFP-GR (A) referred from our previous work [3] and mStayGold-GR (B). Black: eGFP-GR, Red: mStayGold-GR, Green: Gaussian curve fitting. **(C)** Average ± Standard deviation of chi-square value *per* degree of freedom. Two-tailed Student's t-test indicated significant difference between eGFP-GR and mStayGold-GR.

Supplementary references

[1] J. Ries, S. Chiantia, P. Schwille, Accurate determination of membrane dynamics with line-scan FCS, Biophys. J., 96 (2009) 1999.
[2] R. Macháň, Y.H. Foo, T. Wohland, On the Equivalence of FCS and FRAP: Simultaneous Lipid Membrane Measurements, Biophys. J., 111 (2016) 152.
[3] S.N. Nikolić, S. Oasa, A.J. Krmpot, L. Terenius, M.R. Belić, R. Rigler, V. Vukojević, Mapping the Direction of Nucleocytoplasmic Transport of Glucocorticoid Receptor (GR) in Live Cells Using Two-Foci Cross-Correlation in Massively Parallel Fluorescence Correlation Spectroscopy (mpFCS), Analytical Chemistry, 95 (2023) 15171-15179.
